# Structural insights into Parkin substrate lysine targeting from minimal Miro substrates

**DOI:** 10.1038/srep33019

**Published:** 2016-09-08

**Authors:** Julian L. Klosowiak, Sungjin Park, Kyle P. Smith, Michael E. French, Pamela J. Focia, Douglas M. Freymann, Sarah E. Rice

**Affiliations:** 1 Department of Cell and Molecular Biology, Feinberg School of Medicine, Northwestern University, 303 East Chicago Avenue, Chicago, IL 60611, USA; 2 Molecular and Cell Biology Laboratory, Salk Institute for Biological Studies, 10010 North Torrey Pines Road, La Jolla, CA 90237, USA; 3 Department of Biochemistry and Molecular Genetics, Feinberg School of Medicine, Northwestern University, 303 East Chicago Avenue, Chicago, IL 60611, USA

## Abstract

Hereditary Parkinson’s disease is commonly caused by mutations in the protein kinase PINK1 or the E3 ubiquitin ligase Parkin, which function together to eliminate damaged mitochondria. PINK1 phosphorylates both Parkin and ubiquitin to stimulate ubiquitination of dozens of proteins on the surface of the outer mitochondrial membrane. However, the mechanisms by which Parkin recognizes specific proteins for modification remain largely unexplored. Here, we show that the C-terminal GTPase (cGTPase) of the Parkin primary substrate human Miro is necessary and sufficient for efficient ubiquitination. We present several new X-ray crystal structures of both human Miro1 and Miro2 that reveal substrate recognition and ubiquitin transfer to be specific to particular protein domains and lysine residues. We also provide evidence that Parkin substrate recognition is functionally separate from substrate modification. Finally, we show that prioritization for modification of a specific lysine sidechain of the cGTPase (K572) within human Miro1 is dependent on both its location and chemical microenvironment. Activation of Parkin by phosphorylation or by binding of pUb is required for prioritization of K572 for modification, suggesting that Parkin activation and acquisition of substrate specificity are coupled.

Parkinson’s disease (PD) is the second most common neurodegenerative disorder^1^. Mutations in several different genes have been linked to hereditary forms of early-onset Parkinson’s disease, particularly the protein kinase PINK1 and the E3 ubiquitin ligase Parkin; mutations in the genes encoding these two proteins are the leading cause of hereditary Parkinsonism^2^,^3^. Compelling evidence suggests that PINK1 and Parkin function together in a common mitochondrial quality control pathway responsible for the detection and clearance of damaged mitochondria^4^. Healthy mitochondria contain very little PINK1; under basal conditions, Parkin resides in the cytoplasm in an inactive, autoinhibited state^5^,^6^,^7^,^8^,^9^,^10^. Mitochondrial damage results in stabilization of PINK1 on the mitochondrial surface, where it phosphorylates cytoplasmic Parkin at residue Serine 65 (S65) in its ubiquitin-like (Ubl) domain, relieving Parkin autoinhibition^11^,^12^,^13^,^14^,^15^,^16^. PINK1 also phosphorylates ubiquitin (Ub) on the conserved S65 to stimulate Parkin activity^17^,^18^,^19^,^20^,^21^. Together, PINK1 and S65-phosphorylated Parkin (p-S65 Parkin) extensively modify the mitochondrial outer membrane (MOM) with phosphorylated ubiquitin (pUb) chains^4^,^18^,^22^. pUb chains serve as a mitochondrial receptor for further allosteric activation and recruitment of Parkin to the MOM, resulting in a self-amplifying feed-forward loop that ultimately sends mitochondria down the pathway of mitophagy^18^,^22^,^23^,^24^,^25^,^26^.

The E3 ubiquitin ligases comprise a large group of about 600 proteins in humans that recognize a diverse set of targets. RING ligases generally act as scaffolds by binding both Ub-loaded E2 and substrate to facilitate Ub transfer from E2 to substrate, while HECT and Ring-Between-Ring (RBR) ligases are covalently charged with Ub through a catalytic cysteine, transferring Ub directly to the substrate lysine^11^. The E3 ligases in each of these families exploit distinct mechanisms for substrate selection and targeting of lysines for modification^27^. Substrate selection can be mediated via binding domains distinct from the catalytic machinery^28^,^29^. In a subset of E3 ligases, however, specificity appears to be mediated by the ligase catalytic domains themselves^27^. With respect to lysine targeting, some E3 ligases exhibit low selectivity, modifying a ‘ubiquitination zone’ that is conformationally accessible to the catalytic machinery^27^,^30^. Others exhibit high selectivity, targeting specific lysine sidechains over others, and in some cases modifying only one lysine of a target protein^29^,^31^,^32^. The recent structure of a HECT E3 ligase in complex with Ub and substrate suggests that specificity of its catalytic architecture is constrained by formation of a composite binding surface at the interface of the ternary complex^33^. Such specificity can be mediated by binding of specific peptide sequences presented by the substrate target protein^11^,^34^. However, the mechanisms driving ubiquitination specificity are poorly characterized, particularly for the RBR family of E3 ubiquitin ligases of which Parkin is a member^35^,^36^.

Parkin ubiquitinates a great number of proteins throughout the cell, including a multitude of proteins on the MOM^37^. Although many Parkin MOM substrate ubiquitination sites appear to be evolutionarily conserved, no sequence or structural recognition motif has been identified^37^. The massive recruitment of Parkin by pUb chains on the MOM likely contributes to the large number and diversity of Parkin-modified targets, however recent work has shown that pUb is not strictly required for substrate ubiquitination^22^. In the absence of pUb, Parkin is generally unable to synthesize Ub chains, instead favoring direct modification and multi-monoubiquitination of its targets^22^. This behavior suggests a mechanism of primary substrate selection independent of, or preceding, pUb chain formation. However, the determinants of primary Parkin substrate specificity and the contribution of PINK1-mediated Parkin and ubiquitin phosphorylation towards substrate lysine targeting, remain largely unknown.

Human Miro1 and Miro2 (hMiro1, hMiro2, also called RhoT1 and RhoT2) are MOM protein homologs that play a critical role in mitochondrial trafficking^38^,^39^,^40^,^41^,^42^,^43^,^44^. Both associate with Parkin upon mitochondrial depolarization and are ubiquitinated on multiple lysines^18^,^37^,^45^,^46^. There is evidence suggesting hMiro1 is also a substrate of PINK1^39^. hMiro1 protein levels appear to be dynamically regulated by the opposing activities of Parkin and the deubiquitinase USP30 46. As Miro is a key Parkin substrate specifically targeted for proteasomal degradation in the context of mitochondrial damage, we sought to gain insight into Parkin substrate recognition and lysine targeting by examining ubiquitination of hMiro1 and hMiro2. Here, we demonstrate that the cGTPase domain is necessary and sufficient for efficient ubiquitination of either hMiro1 or hMiro2 by p-S65 Parkin. We also find that despite considerable similarities between these two substrates, Parkin modifies hMiro1 much more robustly than hMiro2. As this difference provided an opportunity to gain insight into the mechanisms of Parkin substrate specificity, we undertook an extensive structural and biochemical characterization of these two substrates. Guided by several structures of human Miro domains, we generated a minimal hMiro1 cGTPase substrate that recapitulates Parkin specificity, but which is monoubiquitinated primarily at a single lysine. Targeting of this prioritized residue by Parkin was critically dependent on sidechain position and chemical microenvironment. Finally, we show that S65 Parkin phosphorylation or the presence of conjugatable pUb serve not only to stimulate Parkin activity but also increase selectivity towards the prioritized lysine, K572, of its hMiro1 target. Our results provide a framework for understanding a critical step following activation of the Parkin ubiquitination pathway - the recognition and primary ubiquitination of its substrates.

## Results

### Structures of hMiro1 EF hand and cGTPase domains

**Figure 1:**
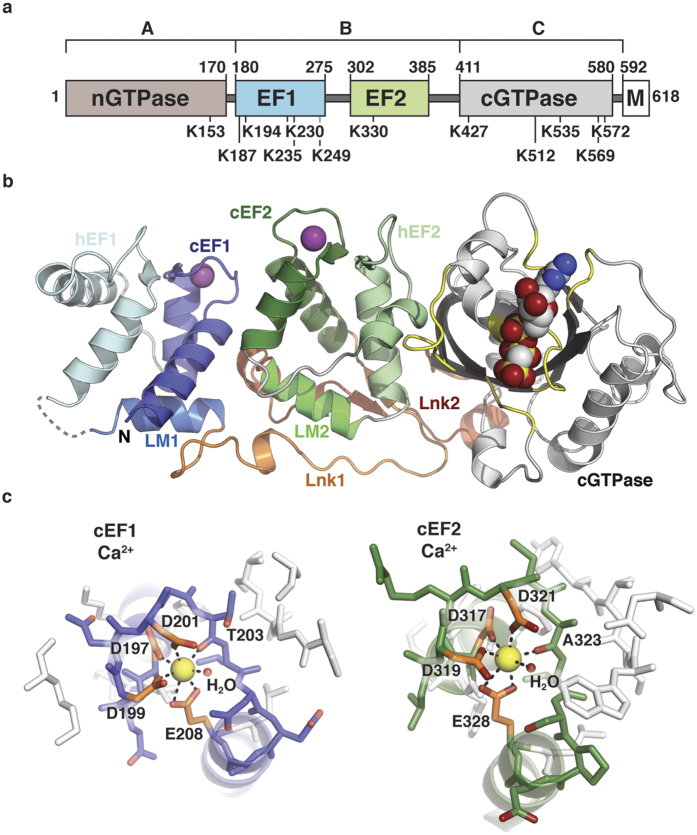
Structure of the hMiro1 EF hand and cGTPase domains. (**a**) Bar diagram of hMiro1 domain architecture. The nGTPase (beige) and cGTPase (gray) flank two Ca^2+^-binding EF hand pairs (blue, EF1; green, EF2); a C-terminal transmembrane domain (M, white) attaches hMiro1 to mitochondria. Parkin-ubiquitinated lysines from all studies to-date^14^,^18^,^37^,^46^ are highlighted below. Numbering corresponds to hMiro1 isoform 3; letters (A, B, C) represent hMiro fragments. (**b**) Crystal structure of hMiro1-BC (aa 177-592). Note three distinct domains: EF1 (blue), EF2 (green), and cGTPase (grey/yellow), joined by linkers Lnk1 (orange), and Lnk2 (red). The structure shown is bound to the non-hydrolyzable GTP analog GMPPCP (CPK representation), with Mg^2+^ bound at both cEF hands (magenta spheres). (**c**) Details of Ca^2+^ coordination by the cEF hands of hMiro1. Ca^2+^ (yellow sphere) bound at cEF1 and cEF2, revealing pentagonal bipyramidal coordination including critical glutamates E208 and E328. Ca^2+^-coordinating residues are labeled. hMiro1, human Miro1; cEF, canonical EF hand; hEF, hidden EF hand; LM, ligand mimic.

We determined the crystal structure of hMiro1 amino acids (aa) 177–592 (hMiro1-BC), encompassing the central EF hand region and cGTPase domain, to 2.5 Å resolution (Fig. 1a,b and Supplementary Table S1). The structure is remarkably similar to the previously published *Drosophila* Miro (dMiro) structure of the same fragment^47^ (53% sequence identity, root mean square deviation (rmsd) 0.94 Å over 329 Cα atoms; Supplementary Fig. S1). We obtained crystals of hMiro1-BC bound to either calcium (Ca^2+^; Fig. 1c), or magnesium (Mg^2+^; Supplementary Fig. S2a), verifying human Miro has two Ca^2+^-binding sites, one at each canonical EF (cEF) hand^42^,^48^. Notably, there was minimal structural change observed between the Ca^2+^-bound vs. Mg^2+^-bound structures, and solution studies showed hMiro1-BC to be monomeric in the presence of either ion (Supplementary Fig. S2b). Similarly, structures of hMiro1-BC bound to either GDP or the non-hydrolyzable GTP analog GMPPCP were virtually superimposable, with GTPase Switch 1 positioned far from the nucleotide-binding pocket in both cases (Supplementary Fig. S2c). Thus, the structure of hMiro1-BC is highly evolutionarily conserved and, in isolation, appears to be conformationally inert with respect to ion or nucleotide binding.

### hMiro1 and hMiro2 are direct primary substrates of Parkin, but are ubiquitinated differently

**Figure 2:**
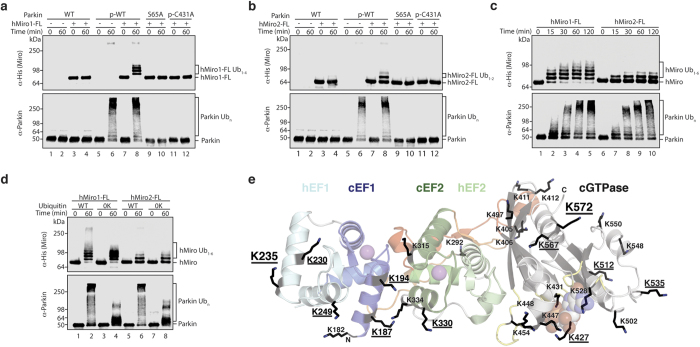
hMiro1 and hMiro2 are direct substrates of p-S65 Parkin, ubiquitinated on multiple lysines. (**a**) hMiro1 is directly ubiquitinated by p-S65 Parkin. 6xHis-tagged full-length hMiro1 (hMiro1-FL, 1 μM) was incubated with various Parkin constructs (0.5 μM): unphosphorylated Parkin (WT), TcPINK1-phosphorylated Parkin (p-WT), and TcPINK1-treated Parkin mutants (S65A, p-C431A), in the presence of E1 (100 nM), E2 (UbcH7, 0.5 μM), and Ub (30 μM). Samples were taken immediately after adding ATP (t = 0 min.) and following incubation at 37 °C (t = 60 min.). Reactions were subjected to immunoblotting to visualize hMiro1-FL (α-His) and Parkin (α-Parkin). (**b**) hMiro2 is directly ubiquitinated by p-S65 Parkin. Reaction conditions as in (a) but with hMiro2-FL. (**c**) p-S65 Parkin ubiquitinates hMiro1-FL more efficiently than hMiro2-FL. Time course assay comparing ubiquitination of hMiro1-FL and hMiro2-FL (2 μM) by p-S65 Parkin (0.5 μM). (**d**) hMiro1-FL and hMiro2-FL are multi-monoubiquitinated by p-S65 Parkin. hMiro1-FL or hMiro2-FL was incubated with p-S65 Parkin in the presence of either wild type (WT) ubiquitin or lysine-less ubiquitin (0 K) incapable of forming ubiquitin chains. hMiro1-FL is efficiently modified at several distinct lysines, as evidenced by the persistence of hMiro1 Ub_1–6_ bands in the 0 K condition. (**e**) The positions of all lysine sidechains of hMiro1 are shown in the context of the hMiro1-BC structure (top-view, rotated 90° relative to Fig. 1b). Of 42 lysines in the primary sequence of hMiro1, 12 have been demonstrated to be ubiquitinated (underlined, save K153 of the nGTPase not present in our structure). Two lysines, K235 and K572 (larger font), are found di-Gly modified in our assays.

Using an *in vitro* reconstitution system consisting of purified human E1 (UBE1), E2 (UbcH7), and Ub, we found that both full-length 6xHis-tagged hMiro1 and hMiro2 are directly ubiquitinated by human Parkin phosphorylated at S65 (p-S65 Parkin; Fig. 2a,b, Supplementary Fig. S3a,b). No ubiquitination was seen using unphosphorylated Parkin, PINK1-treated phosphorylation incompetent Parkin (S65A Parkin), or phosphorylated catalytically inactive (p-C431A) Parkin mutants. These results confirm that S65 phosphorylation releases Parkin from its autoinhibited state and that catalytic activity is strictly dependent upon Parkin’s active site cysteine, C431^7^,^16^,^49^,^50^,^51^ (Fig. 2a,b).

In these experiments, band intensities and band migration positions were reproducible, thereby providing a means for identification of particular modification states, as well as a qualitative measure of ubiquitination levels. We observed that the ubiquitination efficiency of the hMiro homologs was not equal; hMiro1 is converted to ubiquitinated forms more rapidly than hMiro2 (Fig. 2c). We tested whether different ion or nucleotide conditions might influence Miro ubiquitination efficiency by p-S65 Parkin. Using ion or nucleotide exchange protocols similar to those used to obtain crystal structures of hMiro1 constructs in different divalent cation and nucleotide states (see Methods), we were unable to detect a change in hMiro1 or hMiro2 ubiquitination (Supplementary Fig. S4a–c). This result suggests that neither the divalent cations Mg^2+^ and Ca^2+^ nor the nucleotide ligands GDP and GTP directly regulate Parkin-mediated Miro ubiquitination. Similarly, hMiro1 phosphomimetic mutants designed to emulate PINK1 phosphorylation at three reported sites (S156E, T298E, T299E)^39^,^52^ did not increase the efficiency of ubiquitination over wild type levels (Supplementary Fig. S4d). Strikingly, we found that human p-S65 Parkin was able to ubiquitinate dMiro with efficiency comparable to hMiro2, indicating that the elements in hMiro necessary for ubiquitination by Parkin are evolutionarily conserved (Supplementary Fig. S4e,f).

Parkin has been demonstrated to catalyze monoubiquitination at multiple sites *in vitro*^22^,^53^. In order to determine whether the distribution of hMiro1 and hMiro2 Ub conjugates observed in our *in vitro* assays arose from multiple- monoubiquitination by p-S65 Parkin, we carried out ubiquitination reactions with either wild-type ubiquitin (WT Ub) or a lysine-less ubiquitin incapable of chain formation (0 K Ub). These experiments revealed the persistence of a ubiquitin ladder in the 0 K Ub condition, indicating modification at several distinct lysines (Fig. 2d). Mass spectrometric analysis of ubiquitination reactions with WT Ub identified with confidence three diGly-modified lysines in hMiro1 (K153, K235, K572) and hMiro2 (K96, K119, K164) (Supplementary Fig. S5); however, additional sites may have escaped detection in our assays, and previous studies have located Parkin-modified lysines throughout hMiro1 in all four major domains^14^,^18^,^37^,^46^ (Fig. 1a). We have mapped onto the hMiro1-BC crystal structure all hMiro1 lysines that have been reported to be ubiquitinated by Parkin (with the exception of K153, which is in the nGTPase not present in our structure) (Fig. 2e). Lysine K572, uniquely found to be modified in all studies of Parkin-mediated Miro ubiquitination to date^14^,^18^,^37^,^46^, is located within the cGTPase domain along the terminal helix immediately preceding the transmembrane region (Fig. 2e). A conserved lysine at the same position in *Drosophila* Miro, K600, is also modified by human p-S65 Parkin in our assays (Supplementary Fig. S6). The distribution of the remaining modified lysines of hMiro1 does not appear to be random, and is biased towards the two central EF hand pairs. Indeed, 6 of 10 lysine residues in the EF-hands are targeted, whereas only 5 of 18 in the cGTPase domain are modified. While our results show that Parkin clearly does not target lysines within hMiro1 at random, the structure alone does not yield obvious clues as to why certain lysines are ubiquitinated and not others.

### The cGTPase domain is necessary and sufficient for efficient ubiquitination of Miro

**Figure 3:**
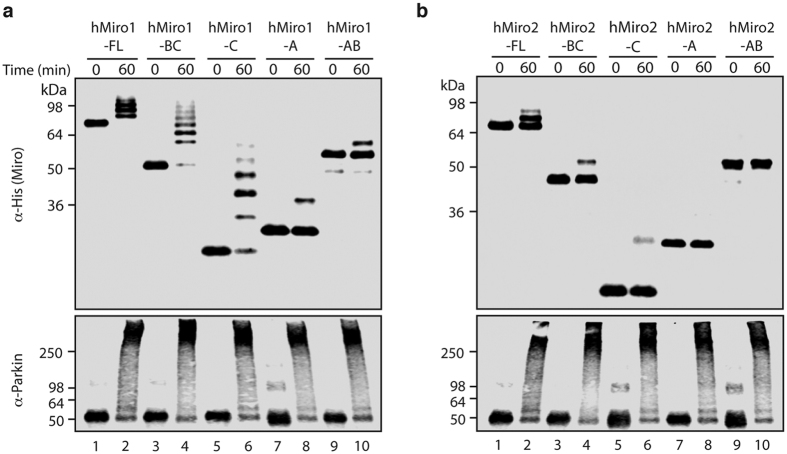
The cGTPase domain of hMiro is necessary and sufficient for efficient ubiquitination by p-S65 Parkin. (**a**) Purified, 6xHis-tagged fragments of hMiro1 (1 μM) were incubated with p-S65 Parkin (0.5 μM) and assessed for ubiquitination (α-His blot). Domains within hMiro1 are labeled as follows: A, corresponding to the nGTPase domain; B, corresponding to the central EF hand region; and C, corresponding to the cGTPase domain (schematized in Fig. 1a). All hMiro1 fragments containing the cGTPase domain were robustly ubiquitinated (hMiro1-FL, hMiro1-BC, hMiro1-C), while the nGTPase alone (hMiro1-A) or the nGTPase with EF hands (hMiro1-BC) showed limited ubiquitination. (**b**) Fragments of hMiro2 incubated with p-S65 Parkin as in (a). hMiro2 fragments containing the cGTPase domain showed evidence of ubiquitination, albeit at reduced levels as compared to hMiro1. hMiro2-A and hMiro2-AB showed no evidence of ubiquitination. Note that hMiro2 has two common allelic variants, R425 and C425; both variants behaved identically in ubiquitination assays (Supplementary Fig. S12) . Parkin activity was equal throughout (α-Parkin blots).

We undertook a domain analysis of Miro in order to determine whether Parkin requires specific regions of Miro for efficient substrate ubiquitination. Guided by the hMiro1-BC crystal structure, we designed a series of truncated 6xHis-tagged domain constructs by dividing hMiro1 and hMiro2 into three regions, corresponding to the nGTPase (A), the central EF hand region (B), and the cGTPase (C), respectively (Fig. 1a). The purified constructs were soluble and well folded (Supplementary Fig. S7a–c). We found that only fragments of hMiro1 containing the cGTPase domain were robustly ubiquitinated by p-S65 Parkin (hMiro1-FL, hMiro1-BC, hMiro1-C) (Fig. 3a). The isolated nGTPase domain (hMiro1-A), or the nGTPase with EF hands (hMiro1-AB), were modified to a much lesser extent, despite harboring lysines that are efficiently ubiquitinated in the full-length protein (Fig. 3a). The addition of hMiro1-BC protein to the hMiro1-A reaction did not rescue ubiquitination in the isolated nGTPase domain *in trans*. However, following introduction of a cleavable linker between a fusion of the hMiro1-C and hMiro1-A domains, there was a specific increase in the ubiquitination efficiency towards the nGTPase domain when fused to the cGTPase *in cis* (Supplementary Fig. S8). This is consistent with the cGTPase domain promoting ubiquitination of the distal nGTPase domain in the context of this artificial domain arrangement.

Fragments of hMiro2 showed a ubiquitination pattern similar to hMiro1, albeit with decreased ubiquitination efficiency and the persistence of unmodified species throughout (Fig. 3b). No modification of hMiro2 fragments lacking the cGTPase was observed (hMiro2-A and hMiro2-AB), while all hMiro2 fragments containing the cGTPase showed ubiquitination (hMiro2-FL, hMiro2-BC, hMiro2-C). In contrast to the isolated hMiro1 cGTPase, however, the isolated cGTPase of hMiro2 (hMiro2-C) was only weakly modified, suggesting the cGTPase domain of hMiro2 may not itself contain efficiently modified lysines. Nevertheless, consistent with our observations in hMiro1, the cGTPase in hMiro2 remains a critical determinant of ubiquitination since hMiro2 is not modified in its absence (Fig. 3b). Taken together, we conclude that the respective cGTPase domains play a role in both substrate recognition and the efficient ubiquitination of lysines throughout both hMiro1 and hMiro2. Further, if the cGTPase domains contribute a required determinant for substrate selection that is absent in the other domains of hMiro1/2, the mechanism of substrate recognition by Parkin must, to some extent, be distinct from the mechanism of substrate ubiquitination.

### hMiro1 and hMiro2 cGTPase structures reveal dimerization

**Figure 4:**
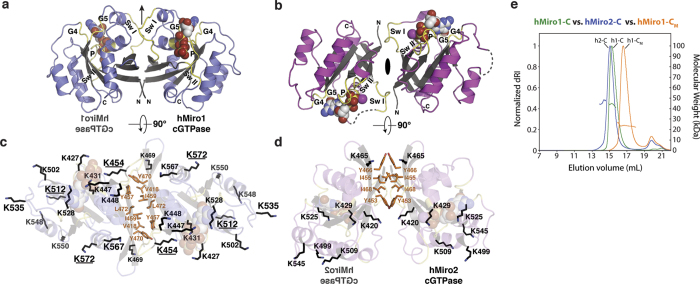
Structures of hMiro1-C and hMiro2-C reveal cGTPase dimerization via a conserved hydrophobic surface. (**a**) Crystal structure of hMiro1-C cGTPase dimer bound to GDP-Pi. Nucleotide binding elements are colored yellow and labeled: P (P-loop); Sw I (Switch I); Sw II (Switch II); G4-G5, guanine nucleotide-binding motifs G4 and G5. Note the two-fold rotational symmetry, highlighted by the mirror image hMiro1 cGTPase label and crystallographic two-fold (arrow). (**b**) Crystal structure of hMiro2-C cGTPase dimer bound to GDP; labels are as in (a). Note the two-fold rotational symmetry (crystallographic dyad). (**c**) Location of lysine side chains (black sticks) and hydrophobic residues (orange sticks) at hMiro1-C dimer interface. Lysines with underlined labels were diGly-modified in a dimeric hMiro1-C ubiquitination sample. (**d**) Location of lysine side chains and hydrophobic residues at hMiro2-C dimer interface. Dimer symmetry is indicated by the mirror image hMiro2 cGTPase label. (**e**) hMiro1-C and hMiro2-C dimerize in solution via their conserved hydrophobic surfaces. SEC-MALS traces of hMiro2-C (blue, left), hMiro1-C (green, middle), and hMiro1-C_M_ with three mutations in its hydrophobic dimer interface (V418R, Y470S, L472A, orange, right). Both hMiro1-C and hMiro2-C elute at twice their calculated molecular weights (hMiro1-C: 21.9 kD; hMiro2-C: 20.2 kD), while hMiro1-C_M_ elutes as a monomer. Typical SEC differential refractive index (dRI) profiles are shown, normalized for each run (y-axis on the left). In-line MALS profiles across each elution peak are shown (y-axis on the right). Concentration of injected proteins: hMiro1-C (16 mg/mL); hMiro2-C (7 mg/mL); hMiro1-C_M_ (18 mg/mL).

To better understand the difference in ubiquitination efficiency between hMiro1 and hMiro2, we first determined the crystal structures of the isolated cGTPase domains of both proteins to high resolution (hMiro1-C: 2.25 Å; hMiro2-C: 1.69 Å; Fig. 4a,b and Supplementary Table S1). The overall folds of the hMiro1 and hMiro2 cGTPase domains are quite similar (rmsd 0.78 Å over 129 Cα atoms; Supplementary Fig. S9a). Unexpectedly, both domains crystallized as symmetric dimers. The dimer interface is a highly conserved hydrophobic surface along one face of the cGTPase domain (Fig. 4c,d), which, in the intact hMiro1-BC structure, forms the interface with the second hidden EF hand (hEF2). The cGTPase does not mediate dimerization in the context of the full-length molecule, as the hEF2/cGTPase interface is mutually exclusive with cGTPase dimerization (Supplementary Fig. S9b,c), and hMiro1-BC is purely monomeric even at high concentrations (Supplementary Fig. S2b).

The residues of the cGTPase domain that interact with hEF2 are structurally conserved in the hMiro1 and hMiro2 homologs, and a structure-based alignment of the four residues of the hMiro1 hEF2 at the core of the hEF2/cGTPase interface shows they are 100% conserved in hMiro2 hEF2 (Supplementary Fig. S9b). Interestingly, although the same conserved residues mediate dimerization in hMiro1-C and hMiro2-C, their dimer relationships differ significantly (Fig. 4a,c, Supplementary Fig. S9d). We performed size exclusion chromatography with in-line multi-angle light scattering (SEC-MALS) and confirmed that both hMiro1-C and hMiro2-C dimerize in solution (Fig. 4e). Subsequently, we successfully disrupted dimerization of hMiro1-C in solution by introducing three mutations at the hydrophobic interface (V418R, Y470S, L472A), thereby generating a stable hMiro1 cGTPase monomer (hMiro1-C_M_; Fig. 4e). The finding that the isolated cGTPases dimerize in solution, and that the dimerization of the hMiro1 cGTPase can be disrupted to generate a stable monomer, proved remarkably useful, as the distinct behavior of lysine targeting in the monomeric hMiro1-C_M_ vs. the dimeric hMiro1-C (hereafter termed hMiro1-C_D_) allowed us to functionally distinguish substrate recognition from the modification of particular lysines on that substrate (below).

### Parkin primarily targets K572 in hMiro1 cGTPase

**Figure 5:**
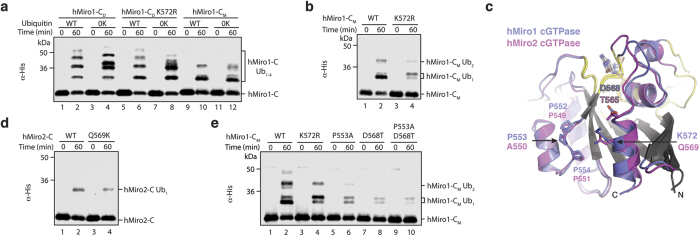
Parkin preferentially targets K572 in the hMiro1 cGTPase domain due to its location and microenvironment. (**a**) Disrupting hMiro1-C dimerization alters the cGTPase ubiquitination pattern. Various hMiro1-C (1 μM) constructs were incubated with p-S65 Parkin (0.5 μM) in the presence of either WT Ub or 0 K Ub (30 μM). hMiro1-C_D_ dimer is efficiently ubiquitinated on multiple lysines, as is hMiro1-C_D_ K572R (lanes 1–8). In contrast, the isolated monomeric hMiro1 cGTPase, hMiro1-C_M_, is primarily monoubiquitinated (lanes 9–12). (**b**) hMiro1-C_M_ is a mostly single-lysine substrate primarily ubiquitinated at K572. hMiro1-C_M_ Ub_1_ travels as a doublet characterized by a dark bottom band. A K572R mutation in hMiro1-C_M_ abolishes this dark bottom band, leaving a faint doublet. (**c**) Structural alignment of the hMiro1 and hMiro2 cGTPases highlights differences in the vicinity of the C-terminal helix. K572 in hMiro1 (blue) corresponds to Q569 in hMiro2 (pink). The chemical environment of the hMiro1 K572 side-chain is also distinct between the two proteins: P553 in hMiro1 vs. A550 in hMiro2, D568 in hMiro1 vs. T565 in hMiro2. (**d**) A Q569K mutation in hMiro2-C does not increase ubiquitination by p-S65 Parkin as compared to wild-type hMiro2-C. (**e**) Targeted ubiquitination at hMiro1 K572 is critically sensitive to chemical microenvironment. Mutation of the hMiro1-C_M_ sequence to corresponding residues of hMiro2-C, P553A and D568T, greatly reduce the efficiency and specificity of ubiquitination. The single and double mutants show no preference for K572 targeting, as evidenced by the Ub_1_ doublet with roughly equal top and bottom band intensities (lanes 6, 8, 10).

Our initial domain dissection experiments confirmed that the cGTPase dimer, though presumably non-physiological, retained its activity as a substrate robustly ubiquitinated by Parkin. Remarkably, although the hMiro1-C_M_ species was also robustly ubiquitinated, it exhibited a different ubiquitination pattern. In particular, hMiro1-C_D_ was robustly ubiquitinated on multiple lysines (Figs 4c and 5a), while hMiro1-C_M_ was primarily monoubiquitinated (Fig. 5a,b). By mass spectrometry we could, nevertheless, identify several residues ubiquitinated in hMiro1-C_M_; however, as K572 is the only lysine that has been identified in all reports of Miro ubiquitination, we suspected that it was the most efficiently ubiquitinated residue of the primarily monoubiquitinated hMiro1-C_M_ species. We tested this idea by introducing the arginine substitution, K572R, into both constructs. Remarkably, we found that overall ubiquitination efficiency of the hMiro1-C_M_ K572R substrate was significantly reduced, while ubiquitination efficiency towards hMiro1-C_D_ K572R was not (Fig. 5a,b). We conclude that the single primary site of efficient modification of the hMiro1-C_M_ monomer is K572, whereas multiple lysines are efficiently targeted in the hMiro1-C_D_ dimer, regardless of whether K572 is present (Fig. 4c).

We exploited the fortuitous observation that the singly-modified species of hMiro1-C_M_ (hMiro1-C_M_ Ub_1_) exhibited both distinct migration patterns and ubiquitination efficiencies, as evidenced by the distinct band intensities of the hMiro1-C_M_ Ub_1_ doublet (Fig. 5b), to identify the major (lower) band of the doublet as arising from prioritized ubiquitination at K572 (Fig. 5b, compare lanes 2 and 4). Thus, our two minimal Parkin substrates exhibit distinct modification behavior – the one for which modification is largely dependent on the presence of a particular lysine sidechain (K572 in hMiro1-C_M_), and the other, structurally similar, for which it is not (hMiro1-C_D_). We note that the two lysines in addition to K572 identified as ubiquitinated by mass spectrometry of the hMiro1-C_D_, K454 and K512, can be mapped in the dimer, but not the monomer, to roughly the same continuous surface containing K572 (Fig. 4c).

### Substrate microenvironment activates hMiro1 K572

We next sought to establish a structural basis for the difference in ubiquitination between hMiro1 and hMiro2, and the preference p-S65 Parkin exhibits for targeting K572 in hMiro1. hMiro2 contains a glutamine, Q569, at the position equivalent to hMiro1’s K572 (Fig. 5c). We tested the role of lysine at that position by introducing a K572R mutation in full-length hMiro1 (hMiro1-FL K572R). We found that hMiro1-FL K572R ubiquitination efficiency is significantly decreased as compared to wild-type hMiro1-FL, such that hMiro1-FL K572R ubiquitination efficiency mirrors that of hMiro2-FL (Supplementary Fig. S10a, compare Fig. 2c). This suggests that hMiro1 K572 may account for the difference in ubiquitination efficiency between hMiro1 and hMiro2. An hMiro2-C Q569K mutation was insufficient to improve ubiquitination, however, showing that the discrepancy between hMiro1 and hMiro2 cGTPase ubiquitination efficiencies does not arise simply because a target lysine is not present at the corresponding location in the hMiro2 structure (Fig. 5d).

Beyond the location of this key target lysine, the electrostatic surface potentials of the cGTPase homologs are also markedly different, especially in the vicinity of K572/Q569 (Supplementary Fig. S10b). The contribution of neighboring hydrophobic and/or acidic residues to the desolvation and deprotonation of acceptor lysines has been demonstrated to facilitate isopeptide bond formation in numerous ubiquitin and ubiquitin-like conjugation systems^11^,^13^. We therefore tested whether the chemical microenvironment of K572 plays a role in activating it for ubiquitin conjugation. Since both hMiro1 and dMiro are ubiquitinated at a lysine in the C-terminal alpha helix but hMiro2 is not, we compared the structures of all three proteins in the vicinity of hMiro1 K572 for hydrophobic and acidic residues structurally conserved between hMiro1 and dMiro but not hMiro2. We identified two such candidate hMiro1 residues: P553 and D568 (Fig. 5c and Supplementary Fig. S6a). P553 is flanked by prolines and lies in a loop immediately adjacent to K572. D568 is found one turn above K572 in the C-terminal alpha helix. Mutation of either of these residues to the corresponding hMiro2 residue (P553A or D568T) generated constructs that were stable and well-folded, demonstrated by SEC-MALS (Supplementary Fig. S7d). Nevertheless, the P553A and D568T mutations, both individually and as the double mutation, completely eliminated K572 substrate lysine prioritization by p-S65 Parkin, evidenced by the resulting equal intensity hMiro1-C_M_ Ub_1_ doublet (Fig. 5e). Curiously, introduction of either also decreased overall ubiquitination levels of hMiro1-C_M_; in contrast, mutation of the targeted lysine itself, K572R, abrogated ubiquitination at that residue but did not significantly change overall ubiquitination levels. These findings demonstrate that neighboring residues contribute to the targeting of particular substrate lysines for modification, and suggest that residues P553 and D568 in particular may be important for overall recognition of the hMiro1 cGTPase by p-S65 Parkin.

### Parkin phosphorylation confers substrate lysine prioritization

**Figure 6:**
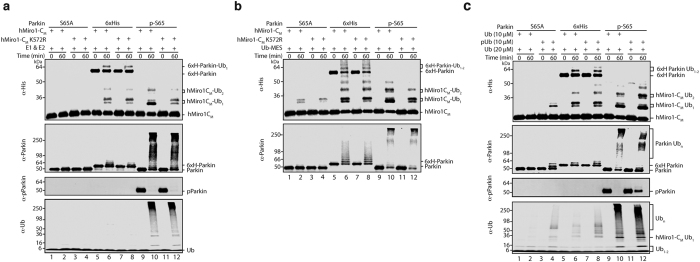
Parkin phosphorylation results in both catalytic activation and substrate lysine prioritization. (**a**) Phosphorylation of Parkin confers the ability to preferentially target K572 in the hMiro1 cGTPase domain. The indicated Parkin constructs (0.5 μM) were incubated with the minimal hMiro1-C_M_ substrate (1 μM) in the presence of E1, E2, and Ub (30 μM). Untagged S65A Parkin is autoinhibited and shows no activity (lanes 1–4). 6xHis Parkin artificially activated with an N-terminal tag results in low levels of substrate and autoubiquitination (lanes 5–8). The hMiro1-C_M_ Ub_1_ doublet pattern reflects no apparent preference for ubiquitination at K572, confirmed by the nearly identical pattern in hMiro1-C_M_ K572R (lane 6 vs. 8). In stark contrast, p-S65 Parkin prioritizes K572 ubiquitination over other cGTPase lysines, (dark bottom hMiro1-C_M_ Ub_1_ doublet band, absent in the hMiro1-C_M_ K572R mutant; lanes 8–12). (**b**) E2 is dispensable for substrate lysine prioritization by p-S65 Parkin. Reaction scheme as in (a), except E1, E2 and Ub were replaced with Ub chemically activated at its C-terminus (Ub-MES; 100 μM). Ub-MES has a mild activating effect on untagged Parkin, generating a weak hMiro1-C_M_ Ub_1_ doublet with evidence for K572 ubiquitination, but not prioritization (lanes 1–4). Ub-MES exerts a robust activating effect on artificially disinhibited 6xHis Parkin, but yields an hMiro1-C_M_ ubiquitination pattern qualitatively identical to untagged Parkin (lanes 5–8). Phosphorylated Parkin is able to efficiently target K572 in the absence of E2 (lanes 9–12). (**c**) Comparison of the effect of pUb on various Parkin constructs. Parkin (0.5 μM) was incubated with hMiro1-C_M_ (1 μM) in the presence of E1, E2, and 30 μM Ub or 20 μM Ub + 10 μM pUb. pUb activates p-S65A Parkin for substrate ubiquitination, autoubiquitination and Ub chain synthesis (lanes 1–4). pUb has a very mild activating effect on artificially disinhibited 6xHis Parkin (lanes 5–8). pUb has a differential effect on p-S65 Parkin, apparently stimulating hMiro1C_M_ ubiquitination while simultaneously suppressing Parkin autoubiquitination and Ub chain synthesis (lanes 9–12).

During the course of experiments characterizing hMiro1-C_M_ as a reporter of targeted ubiquitin transfer, we performed substrate ubiquitination assays comparing three different Parkin constructs: non-phosphorylatable (S65A Parkin), artificially-activated (6xHis Parkin), and fully active phosphorylated Parkin (p-S65 Parkin). S65A Parkin is devoid of activity even after mock-phosphorylation using TcPINK1, consistent with unphosphorylated full-length Parkin existing in an Ubl-mediated autoinhibited state (Fig. 6a, lanes 1–4). N-terminal tags interfere with Parkin autoinhibition^9^,^54^, thereby eliminating the requirement that Parkin be phosphorylated to obtain a degree of catalytic activity. 6xHis Parkin exhibited autoubiquitination and substrate ubiquitination activities (Fig. 6a, lanes 5–8), consistent with a partial release of autoinhibition by the N-terminal 6xHis tag. Surprisingly, the hMiro1-C_M_ ubiquitination pattern in the presence of 6xHis Parkin showed little evidence for prioritization of K572, as the distribution of the hMiro1-C_M_ Ub_1_ doublet band intensity was reversed relative to the much more active p-S65 Parkin. The latter displayed dramatically upregulated autoubiquitination activity and a much higher intensity lower band of the hMiro-1C_M_ Ub_1_ doublet, indicative of efficient ubiquitination of K572 (Fig. 6a lanes 9–12). When tested with the hMiro1-C_M_ K572R mutant, p-S65 Parkin generates an hMiro1-C_M_ Ub_1_ doublet pattern that bears close resemblance to the doublet produced by 6xHis Parkin with hMiro1-C_M_ K572 intact, with a higher intensity upper band corresponding to ubiquitination of other lysines (Fig. 6a lanes 6 vs. 12). These data suggest that, in the absence of prioritization towards K572, the relative activity of both 6xHis Parkin and p-S65 Parkin towards the remaining substrate lysines are roughly equal, despite vastly different levels of activation. That is, activation of Parkin appears to be accompanied by acquisition of specificity for modification of a specific substrate lysine. Further, the absence of prioritization exhibited by the artificially-activated 6xHis Parkin demonstrates that catalytic activity can be uncoupled from substrate lysine prioritization.

Additional support for the latter notion comes from experiments carried out using a so-called “Bypassing System” (ByS) in which a chemically activated ubiquitin is conjugated to mercaptoethanesulfonate at its C-terminus (termed Ub-MES; Fig. 6b)^55^. This approach obviates the need for E1 and E2 by allowing the Ub-MES ubiquitin thioester to undergo direct transthiolation with Parkin’s catalytic cysteine^55^. We found that Ub-MES itself was able to marginally activate S65A Parkin for hMiro1-C_M_ ubiquitination, though no autoubiuquitination was detected (Fig. 6b lanes 1–4). The effect of Ub-MES on the non-physiologically activated 6xHis Parkin was considerably more robust (Fig. 6b lanes 5–8). Remarkably, as in the native cascade, when activated using this Ub-MES bypassing system neither S65A Parkin nor 6xHis Parkin prioritize K572 for modification over other substrate lysines (Fig. 6b lanes 2,6 vs. 4,8). In contrast, similarly activated p-S65 Parkin retained the ability to efficiently target K572 over all other lysines in hMiro1-C_M_ despite the absence of E2 in the reaction (Fig. 6b lanes 9–12).

### pUb also facilitates Parkin substrate prioritization

We next investigated the contribution of pUb to substrate lysine ubiquitination by Parkin. pUb has been found to activate Parkin^17^,^19^,^20^ by a mechanism recently shown to be allosterically coupled to that of Parkin phosphorylation^25^,^26^,^56^. However, pUb binding does not activate Parkin to the same extent as phosphorylation at S65, suggesting a requirement for both for full activity (i.e. pUb-bound p-S65 Parkin)^18^. Conjugatable pUb has an activating effect on unphosphorylated S65A Parkin, triggering ubiquitination of hMiro1-C_M_, autoubiquitination of Parkin, and free Ub chain synthesis to a limited degree (Fig. 6c). Remarkably, when activated by pUb, S65A Parkin prioritized modification of K572, as demonstrated by side-by-side comparison of hMiro1-C_M_ and hMiro1-C_M_ K572R ubiquitination at three different pUb concentrations (Supplementary Fig. S11). When we combined phosphorylated p-S65 Parkin with pUb, the hMiro1-C_M_ ubiquitination pattern was similar to p-S65 Parkin alone (Fig. 6c, lane 10 vs. 12). In contrast, conjugatable pUb apparently had little effect on the behavior of artificially activated 6xHis Parkin (Fig. 6c, lanes 5–8) and there was no evidence for increase in K572 prioritization by 6xHis Parkin in the presence of pUb. This latter result suggests that artificial activation of Parkin by an N-terminal tag, in addition to uncoupling lysine prioritization from activity, also disrupts its intrinsic regulatory mechanisms.

## Discussion

There has been substantial recent progress in understanding the mechanism by which Parkin is released from autoinhibition and activated by phosphorylation and pUb^17^,^25^,^26^,^56^. Also becoming well-characterized is the phenomenology of the role of Parkin in the cell^4^,^12^,^18^. Less clear, yet critical for understanding the Parkin pathway, are the protein-protein interactions that determine which subset of proteins become targets for primary modification. The diversity of substrates and the hundreds of Parkin-modified lysines identified^18^,^37^,^46^ have confounded the determination of the mechanism by which Parkin recognizes its substrates and the question of how particular target lysines are modified remains an important one. Here, we present evidence that illumines this critical step. Our dissection of the Parkin substrates hMiro1 and hMiro2 using biochemical and biophysical approaches identifies the cGTPase domain as a critical determinant of ubiquitination and largely responsible for differences in hMiro isoform ubiquitination efficiency. We provide evidence that Parkin substrate recognition and ubiquitination activity are separable and, by exploiting the discovery of a minimal hMiro-C_M_ substrate, we show that prioritization of a specific primary substrate lysine for ubiquitination, which occurs with physiologically but not artificially activated Parkin, can be uncoupled from Parkin’s catalytic activity. Finally, we demonstrate that elements of the local microenvironment of a targeted lysine residue are critical to prioritization for ubiquitination by Parkin.

That the cGTPase domain of either hMiro1 or hMiro2 is necessary and sufficient for efficient ubiquitination of lysines throughout the entire substrate suggests that Parkin substrate selection is driven first and foremost by a recognition event that, in Miro, occurs within the cGTPase domain. This selection appears to be independent of ubiquitin transfer, as the hMiro2 cGTPase domain does not itself contain preferentially modified lysines and yet remains necessary for the efficient ubiquitination of lysines outside the cGTPase. Thus, substrate recognition by Parkin is uncoupled from substrate modification. This is consistent with the finding that E3 ubiquitin ligases frequently recognize substrates by binding defined sequence/structural motifs and often behave as tethered, modifying lysine residues within a conformationally accessible ‘ubiquitination zone’^11^,^27^,^33^,^57^. The differences in ubiquitination that we observe between the hMiro1-C_D_ dimer and the hMiro1-C_M_ monomer, and the persistence of robust ubiquitination of hMiro1-C_D_ in the K572R mutant, can readily be explained by a model in which Parkin recognizes the cGTPase domain and ubiquitinates K572 “in cis”, but modifies multiple conformationally accessible lysines of the distal domain of the dimer pair “in trans”.

Our data with hMiro1 and hMiro2 also provide additional evidence that, in the absence of pUb, activated p-S65 Parkin multi-monoubiquitinates its substrates, an observation that was recently made in ubiquitin replacement cells^22^. Lysine K572 has been identified as modified in all studies to date, however, numerous other hMiro-FL ubiquitination sites have been identified as well^14^,^18^,^37^,^46^. In the context of full-length Miro, in which the cGTPase forms a conserved interface with the central EF hand region, lysine targeting within the nGTPase and EF hand domains may thus be driven in part by structural constraints imposed by the Parkin/cGTPase interaction. We propose that the population of lysines identified as sites of modification in hMiro-FL may reflect the conformational freedom of either the E3 ligase or its multi-domain hMiro substrate, allowing for modification of a subset of lysines modulated by substrate lysine position and local microenvironment. We have yet to fully reconcile, however, how low efficiency ubiquitination observed during phosphorylation-independent artificial activation of Parkin, whether by an N-terminal 6xHis tag or with Ub-MES, occurs without prioritization of K572.

Although we find that the presence of conjugatable pUb does not fully activate unphosphorylated Parkin for Miro ubiquitination as compared to p-S65 Parkin, pUb alone does confer a degree of specificity to Parkin activity that permits K572 prioritization. This is consistent with data showing chain linkage specificity is similar between pUb-activated Parkin and p-S65 Parkin, but with reduced activity by the former^18^. Importantly, the fact that both S65 phosphorylation and pUb binding enable K572 prioritization by Parkin suggests that the mechanistic pathways for activation by phosphorylation or pUb may partially overlap.

While the structural mechanism by which K572 is prioritized for modification by Parkin in hMiro1 remains unknown, our data support the notion that the chemical microenvironment, as has been suggested for other ubiquitin ligases^33^,^58^, activates the lysine sidechain for nucleophilic attack. Substrate lysine activation is not sufficient for K572 prioritization by Parkin, however. Only S65-phosphorylated Parkin, and to a lesser extent pUb-activated Parkin, efficiently modify K572, while artificially activated Parkin with an N-terminal 6xHis tag or Ub-MES do not. Our experiments with Ub-MES do support the notion that substrate lysine prioritization is inherent to Ub-charged phosphorylated Parkin, independent of the E2/Parkin interaction. Thus, hMiro1 K572 lysine prioritization by Parkin depends upon specific requirements in both substrate and ligase. We speculate that physiological activation of Parkin induces structural changes that facilitate interaction with the cGTPase domain, specifically orienting the Ub-charged active site C431 relative to hMiro1 K572 for efficient, targeted isopeptide ligation. Such a model is in line with crystallographic evidence for a composite ternary complex binding interface in the mechanism of the related HECT E3 ligase Rsp5^33^.

A functional role, if any, for prioritized modification of K572 remains to be established. It is worth noting, however, that beyond its role as a marker for proteolytic degradation, ubiquitination can function as a regulatory post-translational modification^31^,^59^. The biochemistry of the hMiro cGTPase domain is, as of yet, poorly understood^47^. Nevertheless, the conservation of the site between hMiro1 and dMiro and its position on the C-terminal helix of the cGTPase domain suggest the possibility that specific ubiquitination of the Miro cGTPase domain may modulate its function. Although speculative, such an effect would not be unprecedented; indeed, ubiquitination has been demonstrated to impact the functional cycle of the GTPase kRAS, and has been shown to play a regulatory role with respect to function of the MOM GTPase mitofusin^60^,^61^.

Finally, our results suggest that other primary MOM Parkin substrates may also contain subdomains that are specifically recognized by Parkin and that regulate their ubiquitination. These findings carry implications for Parkin substrates that are subunits of multi-protein complexes and/or modified with pUb, as non-covalent association may engender targeted ubiquitin transfer to proteins that do not themselves contain a Parkin “receptor” domain, potentially greatly expanding the repertoire of the Parkin-dependent ubiquitylome while maintaining a degree of specificity^37^. Further work is certainly necessary to fully understand the conformational rearrangements driven by Parkin phosphorylation and the structural basis for the resulting lysine specificity. The results we report here provide an initial framework for investigating the structural biology of this fundamentally important signaling and regulatory behavior.

## Methods

### DNA constructs

Full-length human Miro1 (aa 1-572, isoform 3, C-terminal 6xHis tag in a pET28a(+) vector; hMiro1-FL) was a gift from the Schwarz lab; full length human Miro2 (C425 allelic variant) in a mammalian expression vector, a gift from the Shaw lab, was subcloned into a pET28a(+) vector (aa 1-588, C-terminal 6xHis tag; hMiro2-FL). Residue numbers for hMiro constructs were as follows: hMiro1-BC (177-592), hMiro1-C (411-592), hMiro1-A (1-180), hMiro1-AB (1-417), hMiro2-BC (177-588), hMiro2-C (409-588), hMiro2-A (1-180), hMiro2-AB (1-415), hMiro1-A-PreScission-hMiro1-C (HA tag 1-180-LEVLFQGPGMG-411-592 6xHis tag), hMiro1-AB-hMiro2-C (1-413; 411-588), hMiro2-AB-hMiro1-C (1-411; 413-592). *Drosophila* Miro constructs were as described previously^47^. The construct for full-length human Parkin with an N-terminal 6xHis-SUMO-HA tag was obtained from the University of Dundee, as was the MBP-tagged TcPINK1 construct. Other reagents are as described in Supplementary Methods.

### Protein purification

Details of protein expression are described in Supplementary Methods. For purification of all hMiro constructs from bacteria, Miro lysis buffer consisted of: 25 mM HEPES at pH 7.4, 300 mM NaCl, 0.5 mM TCEP, 8 mM imidazole, 1 mM MgCl_2_, 5% sucrose, 0.02% Tween, 1 mM PMSF, 2 μg/mL Aprotinin, 4.7 μg/mL Leupeptin, 0.7 μg/mL Pepstatin A. Cells were thawed in a 37 °C water bath, then all further steps were conducted at 4 °C unless otherwise noted. Cells were lysed by sonication and pelleted at 35,000 rpm for 45 min in a Ti45 rotor. The cleared lysate was incubated with TALON beads for 1 h, washed using Miro lysis buffer supplemented with 12 mM imidazole, and eluted using Miro lysis buffer supplemented with 300 mM imidazole. Fractions were pooled, diluted ~four-fold with buffer A (25 mM HEPES at pH 7.4, 0.5 mM TCEP, 5% sucrose), loaded onto a HiTrap Q HP anion-exchange column, and eluted with a linear salt gradient (buffer A + 1 M NaCl). All hMiro1-C construct derivatives were purified using a HiTrap S HP cation-exchange column, rather than HiTrap Q. hMiro1-BC was purified by serial HiTrap S then HiTrap Q columns. When necessary, a final purification step on a Superdex 200 (S200) 16/60 size exclusion column was performed in S200 buffer (25 mM HEPES at pH 7.4, 0.5 mM TCEP, 300mM NaCl). Purity was assessed by SDS-PAGE, fractions were pooled and supplemented with 20% sucrose, cleared of aggregates by ultracentrifugation at 100,000 rpm for 10 min, flash frozen in LN_2_ and stored at −80 °C. dMiro was purified as described previously^47^.

MBP-TcPINK1 was purified as described previously^62^. Briefly, lysis buffer consisted of: 1 × PBS, 15 mM imidazole, 1 mM EDTA, 1 mM EGTA, 1% (v/v) Triton X-100, 0.1% (v/v) 2-mercaptoethanol and 0.1 mM PMSF 1 mM Benzamidine. Cells were lysed by sonication and the cleared lysate incubated with amylose beads. Beads were washed with lysis buffer and eluted with 50 mM Tris-HCl (pH 7.6), 150 mM NaCl, 0.1 mM EGTA, 0.25% (v/v) Triton X-100, 0.1% (v/v) 2-mercaptoethanol, 10% glycerol, and 10 mM Maltose. Eluted protein as dialyzed overnight into 50 mM Tris-HCl (pH 7.6), 150 mM NaCl, 0.1 mM EGTA, 0.25% (v/v) Triton X-100, 0.1% (v/v) 2-mercaptoethanol and 10% glycerol, then flash frozen in LN_2_ and stored at −80 °C.

For purification of all untagged Parkin constructs, Parkin lysis buffer consisted of: 75 mM Tris pH 7.5, 500 mM NaCl, 0.2% Triton X-100, 25 mM imidazole, 0.5 mM TCEP, 10 μg/mL DNase, 1 mM benzamidine and 0.1 mM PMSF. Cells were lysed by sonication and the clarified lysate was incubated with Ni-NTA beads for 30 min at 4 °C. The beads were washed with Parkin buffer (25 mM HEPES pH 8.0, 200 mM NaCl and 0.5 mM TCEP) and protein was eluted with wash buffer supplemented with 300 mM imidazole. Eluate was concentrated and loaded onto a Superdex 75 (S75) 16/60 size exclusion column equilibrated in Parkin buffer. 6xHis-SUMO Parkin was then combined with 6xHis-SENP1 at a molar ratio of ~10:1 and incubated for 2 hours at room temperature. After filtering the reaction through fresh Ni-NTA beads to remove 6xHis-SUMO, uncleaved Parkin and 6xHis-SENP1, untagged Parkin was again purified on the S75. Fractions containing pure Parkin as assessed by SDS-PAGE were concentrated, supplemented with 10% glycerol, flash frozen in LN_2_ and stored at −80 °C.

### Parkin and ubiquitin phosphorylation

Full-length, untagged Parkin to be phosphorylated was incubated with TcPINK1 at a molar ratio of ~4:1 for 4 hours at 30 °C in kinase buffer (50 mM Tris-HCl pH 7.6, 10 mM MgCl_2_, 5 mM ATP, 0.1 mM EGTA, 0.5 mM TCEP) prior to SENP1 cleavage. 6xHis-SENP1 was then directly added to the kinase reaction and purification proceeded as described above for untagged Parkin. Ubiquitin was phosphorylated as described previously^18^. Briefly, ubiquitin was incubated with TcPINK1 at a molar ratio of 5:1 for 24 hours at 30 °C in kinase buffer then loaded onto an S75 column equilibrated in 25 mM Tris-HCl pH 7.6, 200 mM NaCl, 0.5 mM TCEP. Eluted pUb was buffer exchanged into water with a PD10 desalting column, loaded onto a HiTrap Q HP anion-exchange column and eluted with a linear gradient of 50 mM Tris-HCl pH 7.6. Parkin and ubiquitin phosphorylation were confirmed by SDS-PAGE using PhosTag (Wako Chemicals), and by MS/MS (p-Parkin) and intact mass analysis (p-Ub) (Supplementary Fig. S3).

### Ub-MES synthesis

Human ubiquitin (Boston Biochem) at 100 μM was incubated with 0.5 μM mouse E1 (purified as described previously^63^) in buffer containing: 50 mM NaPO_4_ pH 8.0, 10 mM MgCl_2_, 5 mM ATP, and 100 mM sodium 2-mercaptoethanesulfonate. A total reaction volume of 23.4 mL (20 mg ubiquitin) was incubated in a 50 mL conical at 37 °C for 5 hours without shaking, then concentrated to ~ 5 mL in a 3 K MWCO spin concentrator and purified over an S75 column equilibrated in 12.5 mM HEPES pH 6.7, 25 mM NaCl. Eluted protein was flash frozen in LN_2_ and stored at −80 °C. Chemical modification was confirmed by intact mass analysis.

### *In vitro* ubiquitination assays

*In vitro* ubiquitination reactions were typically carried out in a 50-μL reaction volume in assay buffer (25 mM Tris pH 7.5, 50 mM NaCl, 6 mM MgCl_2_, 5 mM ATP, and 0.2 mM TCEP) at 37 °C using E1 (Ube1, 100 nM), E2 (UbcH7, 0.5 μM), ubiquitin (30 μM), Parkin (0.5 μM) and Miro (1 μM). Reactions were prepared on ice and initiated with the addition of 10x assay buffer containing ATP, followed by brief mixing, withdrawal of the zero time point, and transfer to a 37 °C water bath. All aliquots were removed at the indicated times, quenched by the addition of 6x Laemmli sample buffer, and analyzed by SDS-PAGE and immunoblotting with the indicated antibody.

For the Ub-MES experiments described in Fig. 6b, reactions were carried out in a 50-μL volume in Ub-MES buffer (25 mM HEPES pH 7.5, 50 mM NaCl, 6 mM MgCl_2_, and 0.2 mM TCEP) at 37 °C using Ub-MES (100 μM), Parkin (0.5 μM) and Miro (1 μM). The Ub-MES reaction described in Supplementary Fig. S11 contained 100 μM Ub-MES, 1 μM Parkin, and 10 μM Miro. Reactions described in Fig. 6c and Supplementary Fig. S11 contained a total of 30 μM ubiquitin, with various proportions of WT Ub to pUb, as indicated.

Our initial hMiro2 construct contained a common hMiro2 allelic missense variant within the P-loop of the cGTPase domain, R425C, which has a minor allelic frequency of 33% (Uniprot: VAR_026638, ensemble: rs3177338). We therefore tested the more common hMiro2 R425 variant, noting that hMiro1 has a conserved lysine at the equivalent position (K427; Supplementary Fig. S1a). However, a side-by-side comparison of R425 and C425 hMiro2 variants reveals no difference, indicating this missense mutation is neutral with respect to Parkin ubiquitination (Supplementary Fig. S12).

### Crystallization and structure determination

Crystals were grown using the sitting-drop vapor diffusion method at 21 °C with a drop size of 1 μL using a Phoenix robot (Art Robbins Instruments). Protein buffer solution contained: 25 mM HEPES, pH 7.4, 300 mM NaCl, 0.5 mM TCEP. For hMiro1-BC, Mg^2+^ and Ca^2+^ exchange was achieved by protein pre-incubation with protein buffer solution supplemented with 1 mM EGTA, followed by the addition of either 5 mM MgCl_2_ or CaCl_2_ and desalting into protein buffer solution supplemented with 5 mM Mg^2+^ or Ca^2+^. For hMiro1-BC nucleotide exchange, protein was pre-incubated with protein buffer solution supplemented with 1 mM EDTA, then incubated with 10 mM GDP or GMPPCP, applied to a desalting column and eluted into buffer containing 1 mM GDP or GMPPCP and 6 mM MgCl_2_. Crystallization conditions were as follows: **hMiro1-BC_Ca**^**2+**^ (10 mg/mL + 5 mM CaCl_2_ + 1 mM GDP): 0.04 M Potassium phosphate, 16% (w/v) PEG 8000, 20% (v/v) glycerol. **hMiro1-BC_GDP** (9 mg/mL + 5 mM MgCl_2_ + 1 mM GDP): 0.2 M Ammonium sulfate, 0.1 M tri-sodium citrate pH 5.6, 25% (w/v) PEG 4000. **hMiro1-BC_GMPPCP** (12 mg/mL + 6 mM MgCl_2_ + 10 mM GMPPCP): 0.2 M Ammonium sulfate, 0.1 M tri-sodium citrate pH 5.6, 17.5% (w/v) PEG 4000; **hMiro1-C_C222**_**1**_ (15 mg/mL + 6 mM MgCl_2_): 0.1 M Tris pH 8.5, 12% (w/v) PEG 4000. **hMiro1-C_P4**_**1**_**2**_**1**_**2** (12 mg/mL): 0.1 M Tris pH 8.5, 16% (w/v) PEG 10000. **hMiro1-C_P3**_**1**_**21** (19 mg/mL + 5 mM MgCl_2_): 0.1 M SPG buffer pH 5, 25% (w/v) PEG 1500. **hMiro2-C** (14 mg/mL): 0.2 M Magnesium Formate, 20% (w/v) PEG 3350. All crystals were harvested directly from their mother liquor without added cryoprotectants.

Measurement of X-ray diffraction data was performed at the beamlines of the Life Sciences Collaborative Access Team (LS-CAT) at Sector 21 of the Advanced Photon Source in the Argonne National Laboratory. Data were measured at 100 K using a MarMosaic 225 CCD detector and were processed using the CCP4 suite^64^. Molecular replacement was carried out in Phenix^65^ using fragments of the *Drosophila* Miro structure as search models (PDB ID 4C0L). Structures were interpreted and rebuilt using COOT^66^. Crystallographic statistics for all structures are presented in Supplementary Table S1. All figures were created in PyMol^67^, Microsoft Excel, and/or Adobe Illustrator. The electrostatic surface potential representations (Supplementary Fig. S10b) were generated using the PyMol APBS plugin^68^.

### SEC-MALS assays

Solution size exclusion chromatography with multi-angle light scattering (SEC-MALS) experiments were conducted using Agilent Technologies 1200 LC HPLS system equipped with a Wyatt Dawn^®^ Heleos™II 18-angle MALS light scattering detectors, Optilab^®^ T-rEX™ (refractometer with EXtended range) refractive index detector, WyattQELS™ quasi-elastic (dynamic) light scattering (QELS) detector and ASTRA software. Proteins were buffer exchanged into SEC-MALS buffer (25 mM HEPES at pH 7.4, 200 mM NaCl, 0.5 mM TCEP, 1 mM MgCl_2_) using an S200 10/300 GL column, spin concentrated, and cleared of aggregates by ultracentrifugation. A total of 200 μL of protein was injected and run on the S200 column at a flow rate of 0.5 mL/min in SEC-MALS buffer at 10 °C. hMiro1-BC samples for which the effect of Ca^2+^ was examined (Supplementary Fig. S2), SEC-MALS buffer was supplemented with either 0.5 mM EGTA or 3 mM CaCl_2_. Bovine serum albumin (BSA) was run as a control in both Ca^2+^-free and 3 mM Ca^2+^ SEC-MALS buffers, and a void volume of 7.8 mL was determined using blue dextran.

## Additional Information

**Accession codes:** Coordinates and structure factors have been deposited in the RCSB protein data bank (PDB) under accession codes: 5KTY (hMiro1-BC_Ca^2+^), 5KU1 (hMiro1-BC_GDP), 5KSZ (hMiro1-BC_GMPPCP), 5KSP (hMiro1-C C222_1_), 5KSY (hMiro1-C P4_1_2_1_2), 5KSO (hMiro1-C P3_1_21), 5KUT (hMiro2-C).

**How to cite this article**: Klosowiak, J. L. *et al*. Structural insights into Parkin substrate lysine targeting from minimal Miro substrates. *Sci. Rep*. **6**, 33019; doi: 10.1038/srep33019 (2016).

## Supplementary Material

Supplementary Information
