# Supplementary material for: Structural insights into Parkin substrate lysine targeting from minimal Miro substrates
Source: Sci Rep. 2016 Sep 8;6:33019. doi: 10.1038/srep33019 (PMC5015425; doi:10.1038/srep33019)
Supplement: Supplementary Information [file srep33019-s1.pdf]

SUPPLEMENTARY INFORMATION FOR:

Structural insights into Parkin substrate lysine targeting from minimal Miro substrates

Julian L. Klosowiak[1], Sungjin Park[1], Kyle P. Smith[1], Michael E. French[3], Pamela J. Focia[2], Douglas M. Freymann[2*], Sarah E. Rice[1*]

[1]Department of Cell and Molecular Biology, [2]Department of Biochemistry, Feinberg School of Medicine, Northwestern University, 303 East Chicago Avenue, Chicago, IL 60611, USA; [3]Molecular and Cell Biology Laboratory, Salk Institute for Biological Studies, 10010 North Torrey Pines Road, La Jolla, CA 90237, USA

*Correspondence: s-rice@northwestern.edu, freymann@northwestern.edu

Supplementary Methods

Protein expression. All hMiro constructs were expressed in *E. coli* BL21-CodonPlus® (DE3)-RP cells cultured in TPM medium containing 50 µg/mL kanamycin and 34 µg/mL chloramphenicol at 37 °C to OD_{600} = ~0.5, cooled to 18 °C and induced with 125 µM IPTG at an OD_{600} = ~0.9 for ~16 h. MBP-TcPINK1 was expressed in *E. coli* BL21-CodonPlus® (DE3)-RIL cells cultured in LB medium containing 50 µg/mL carbenicillin and 34 µg/mL chloramphenicol at 37 °C to OD_{600} = ~0.4, cooled to 16 °C and induced with 250 µM IPTG at an OD_{600} = ~0.7 for ~16 h. 6xHis-SUMO Parkin was expressed in *E. coli* BL21-CodonPlus® (DE3)-RIL cells cultured in LB medium containing 50 µg/mL carbenicillin and 34 µg/mL chloramphenicol at 37 °C to an OD_{600} = ~0.4, cooled to 15 °C, induced with 10 µM IPTG and supplemented with 250 µM $ZnCl_2$ at an OD_{600} = ~0.7 for ~16 h. Cells were harvested by centrifugation, re-suspended in lysis buffer, frozen in liquid nitrogen (LN_2) and stored at – 80 °C.

Other reagents. Anti-polyHistidine antibody (1:2000) was from Sigma. Anti-Parkin antibody (1:2000) was from Santa Cruz. Anti-Parkin (phospho S65) antibody (1:1000) and anti-HA antibody (1:2000) were from Abcam. Anti-ubiquitin antibody (1:1000) was from Cell Signaling. Anti-ubiquitin (phospho S65) antibody (1:750) was from EMD Millipore. Donkey anti-mouse and donkey anti-rabbit antibodies (1:4000) were from LI-COR Biosciences. E1 (Ube1; E-306), E2 (UbcH7; E2-640), Ub (U-100H) were from Boston Biochem. N-terminally 6xHis-tagged Parkin was from Millipore. PreScission protease was from GE Healthcare.

Mass spectrometry. Ubiquitination reactions conducted for the purposes of mass spectrometric identification of di-Gly-modified lysines were carried out using 100 nM E1, 0.5 µM UbcH7, 30 µM ubiquitin, 0.5 µM p-S65 Parkin, and 2 µM Miro for 2h at 37 °C with the

exception of hMiro1-C_M, which was conducted using 200 nM E1, 1 μM UbcH7, 100 μM ubiquitin, 1 μM p-S65 Parkin, and 10 μM hMiro1-C_M to improve detection sensitivity of weakly-modified lysines besides K572. All ubiquitination reactions with Miro subsequently analyzed by mass spectrometry were conducted with Miro in the Mg^{2+}GDP-bound state. Reactions were concentrated in vaccuo, solubilized in 8M urea and reduced with 1mM DTT. The sample was digested overnight at 37° C by diluting from 8M to 1M urea using 100mM ammonium bicarbonate and trypsin. Digested samples were desalted using reverse phase C18 spin columns, concentrated to dryness in vaccuo, re-suspended in 5% acetonitrile and 0.1% formic acid, loaded directly onto a 15 cm, 75 μM reversed phase capillary column (ProteoPep™ II C18, 300 Å, 5 μm size) and separated with a 200-minute gradient from 5% acetonitrile to 100% acetonitrile on a Proxeon Easy n-LC II. The peptides were directly eluted into an LTQ Orbitrap Velos mass spectrometer with electrospray ionization flow rate at 350 nl/minute. Mass spectrometer was operated in data dependent mode. For each MS1 precursor ion scan, the ten most intense ions were selected from fragmentation by CID (collision induced dissociation). Resolution of MS1 was set at 60,000, normalized collision energy 35%, activation time 10 ms, isolation width 1.5, and +4 and higher charge states were rejected. The data were processed using Proteome Discoverer Version 1.4 and searched using embedded SEQUEST HT search engine. Other parameters included: fixed modification: cysteine carbamidomethylation; variable modification: methionine oxidation and ubiquitination on lysine; precursor mass tolerance ±10 ppm; fragment ion mass tolerance ±0.8 Da. The peptide identification was considered valid at q value < 0.1 and each modified peptides were further manually verified for the ubiquitin modification.

Ubiquitination reactions under various ion and nucleotide conditions. The ubiquitination reactions described in Supplementary Figure S4 were conducted as follows: For Mg^{2+} vs. Ca^{2+} reactions, Miro was incubated with 1 mM EGTA for 1h at 4 °C, applied to a mini desalting column and eluted into buffer containing either 5 mM $MgCl_2$ or 5 mM $CaCl_2$. For GDP vs. GMPPCP reactions, Miro was first incubated with 1 mM EDTA for 1h at 4 °C, then incubated with 10 mM GDP or GMPPCP, applied to a mini desalting column and eluted into buffer containing 1 mM GDP or GMPPCP and 6 mM $MgCl_2$.

PreScission ubiquitination experiments. The PreScission ubiquitination experiments described in Supplementary Figure S8 were conducted as follows: For cleavage post-Ub rxn, at t = 60 min 10 units of PreScission protease was added to the reaction, followed by incubation at 37 °C for an additional 30 min prior to reaction termination. For cleavage pre-Ub rxn, h1A/PreScission/h1C was incubated with 10 units of PreScission protease for 30 min at 37 °C, followed by addition to the remaining reaction components at t = 0 min and reaction termination at t = 60 min.

Terbium luminescence. $TbCl_3$ stock was made up at 5mM in H_2O. Protein samples were diluted to 0.5uM in 25 μM $TbCl_3$, 0.1 M Tris 8.0, and aliquoted in duplicate (200 μL) to a Greiner black flat-bottom 96-well microplate. Tryptophan FRET excitation of terbium luminescence was measured using a Tecan Safire 2 microplate reader. Samples were excited at 295nm (10nm bandwidth) and the emission scan carried out over 400-564nm (2nm step, 10nm bandwith), with readings initiated 100 μs after illumination. The data were corrected for protein-free Tb^{3+} background luminescence, yielding the curves shown in Supplemental Fig. S7c. The protein-bound luminescence signal was shown to be Ca^{2+}-sensitive (i.e. consistent

with binding at the cEF2 Ca^{2+} site) by its disappearance following addition of 10mM $CaCl_2$ to the sample.

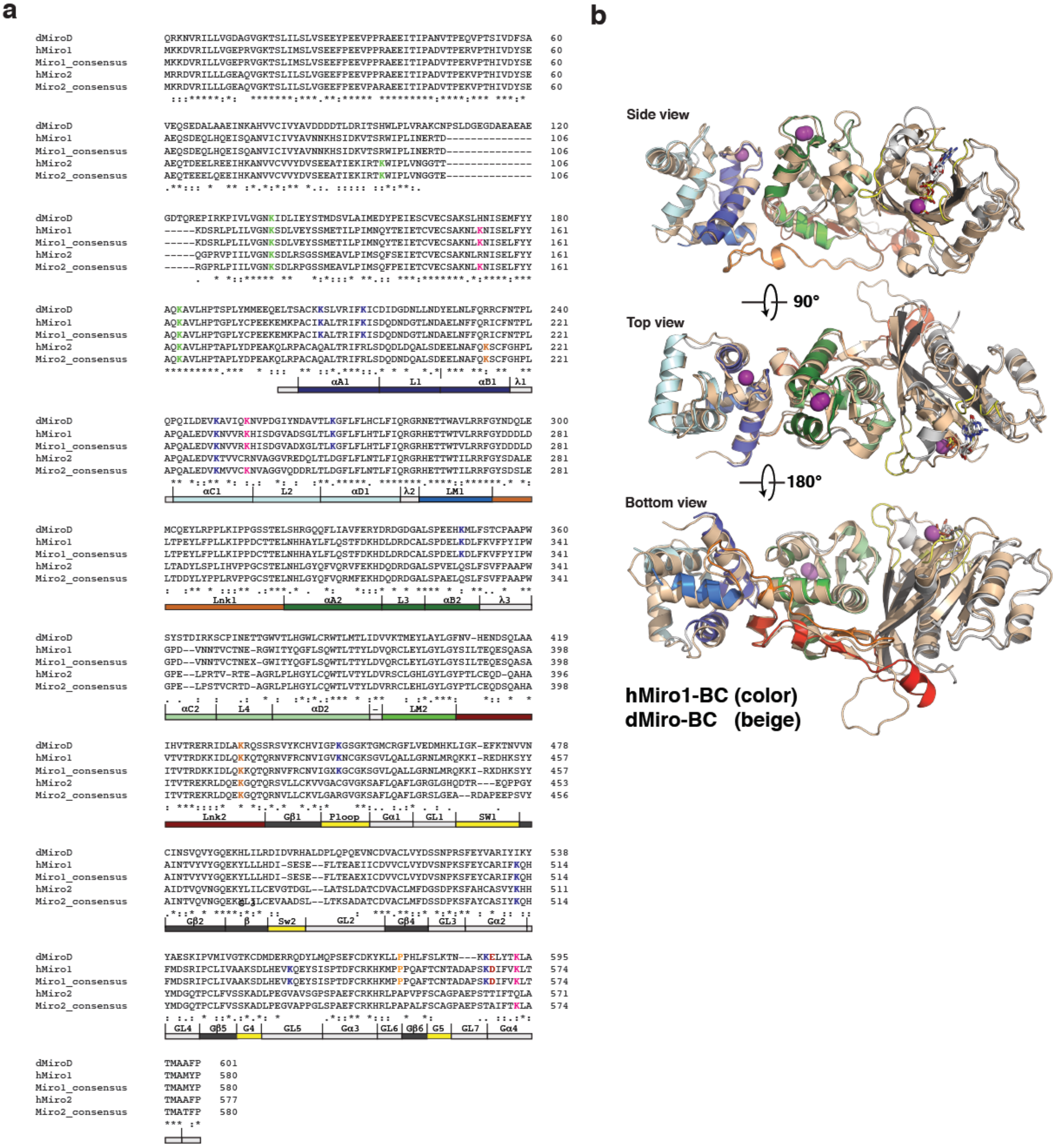

Supplementary Figure 1 | Sequence and structure alignment of Miro. (**a**) Sequence alignment of dMiro, hMiro1, Miro1 vertebrate consensus, hMiro2 and Miro2 vertebrate consensus. Secondary structure based on the hMiro1 crystal structure is shown below the

sequence. Ubiquitinated hMiro1 lysines identified by mass spectrometry in this study, and their conserved counterparts, are in pink; all other hMiro1 lysines identified to be ubiquitinated in previous studies are in blue. Ubiquitinated hMiro2 lysines identified by mass spectrometry in this study, and their conserved counterparts, are in green; all other hMiro2 lysines identified to be ubiquitinated in previous studies are in brown. P553 in hMiro1 is highlighted in orange. D568 in hMiro1 is highlighted in red. "*" indicates identity; ":" and "." indicate similarity, "-" indicates a gap. Sequence reference numbers: hMiro1 (NCBI NP_060777.3); hMiro2 (GenBank CAD56957.1); dMiro (GenBank AAN13971.1). (**b**) Overlay of dMiro-BC and hMiro1-BC crystal structures. The structures are very similar (rmsd 0.942 Å over 329 of 360 Cα atoms), with the exception of Lnk2 (red), which forms a short alpha helix in hMiro1 but is without secondary structure in dMiro. Colors for hMiro1 are as described in Fig. 1; dMiro-BC is shown in beige. Side view is as shown in Fig. 1b, top view is as shown in Fig. 2e. Sequence alignments were made using Clustal Omega with a Needleman-Wunsch algorithm (http://www.ebi.ac.uk/Tools/msa/clustalo/). Sequence statistics were derived from alignments generated by NCBI Protein BLAST (http://blast.ncbi.nlm.nih.gov/Blast.cgi). Structural alignments were generated in PyMol using the "align" command.

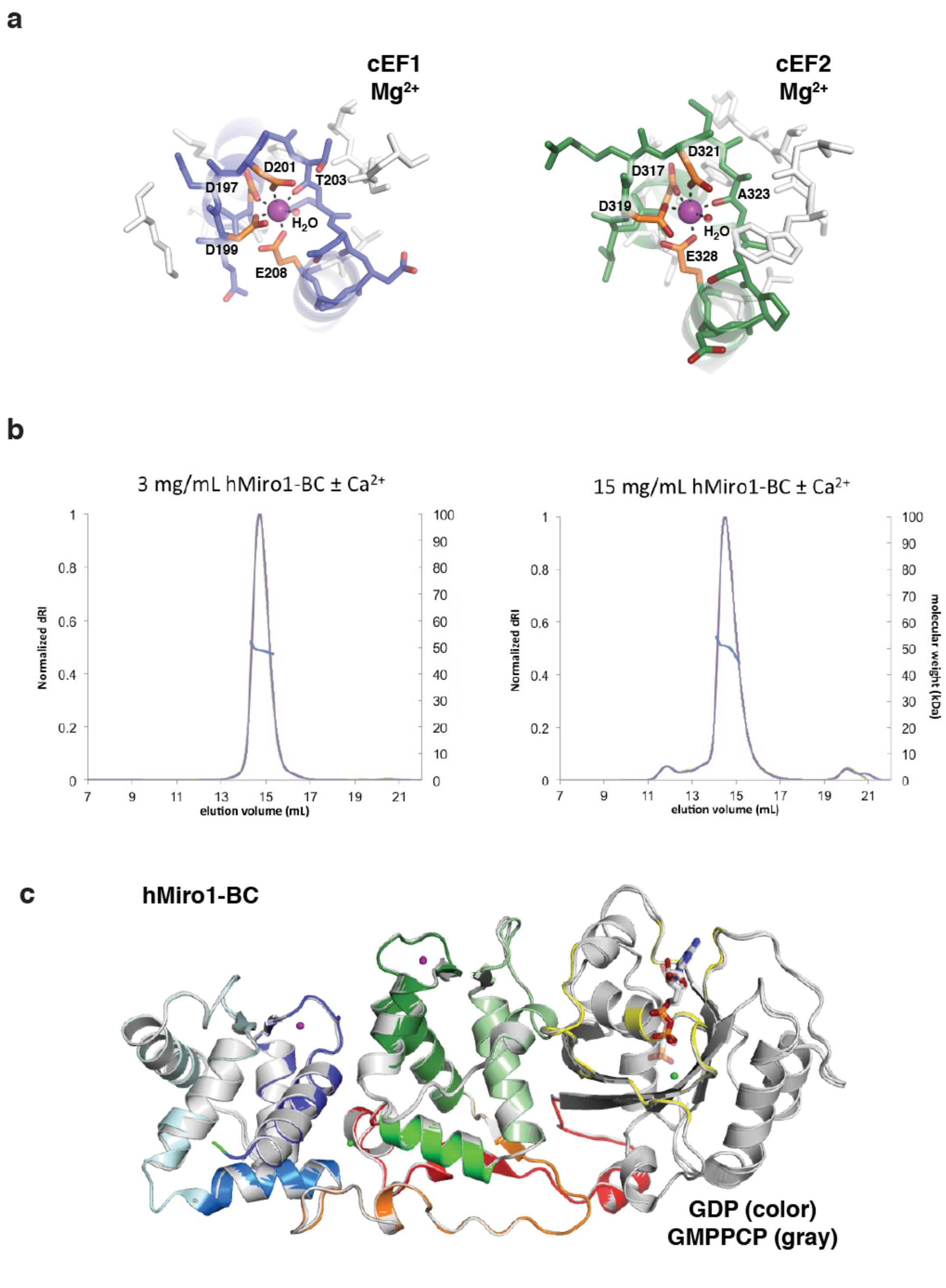

Supplementary Figure 2 | No direct structural effect of cation or nucleotide binding to hMiro1. (**a**) Details of Mg^{2+} coordination by the cEF hands of hMiro1. Mg^{2+} bound to cEF1

(blue, left) and cEF2 (green, right) exhibits the classic octahedral coordination geometry, with the critical glutamate (E208, cEF1; E328, cEF2) contributing only one coordinating bond, and otherwise minimal structural rearrangement compared to the Ca^{2+} bound state (Fig. 1c). Mg^{2+}-coordinating residues are labeled. (**b**) hMiro1-BC (aa 177-592) is a monomer in solution ± Ca^{2+}. Overlay of SEC-MALS traces obtained using 3 mg/mL injected protein (left) and 14.6 mg/mL injected protein (right), each carried out in the presence of either 0.5 mM EGTA (blue/purple) or 3 mM Ca^{2+} (green/red). Typical SEC differential refractive index (dRI) profiles are shown, normalized for each run (y-axis on the left). In-line MALS profiles across each elution peak are shown (y-axis on the right). hMiro1-BC migrates at its predicted molecular weight of 49kD. Note the perfect overlap in both experiments; hence, the traces in each panel are indistinguishable except at high elution volume. (**c**) Overlay of GDP- and GMPPCP-bound hMiro1-BC domains. The structure of hMiro1 is essentially unchanged whether GDP or GMPPCP are bound at the cGTPase domain (rmsd 0.24 Å over 336 of 392 Cα atoms). The positions of Mg^{2+} ions (magenta spheres) and provisionally identified Cl^- ions (green spheres) are indicated. The GDP-bound structure is shown in color; the GMPPCP-bound structure is shown in gray.

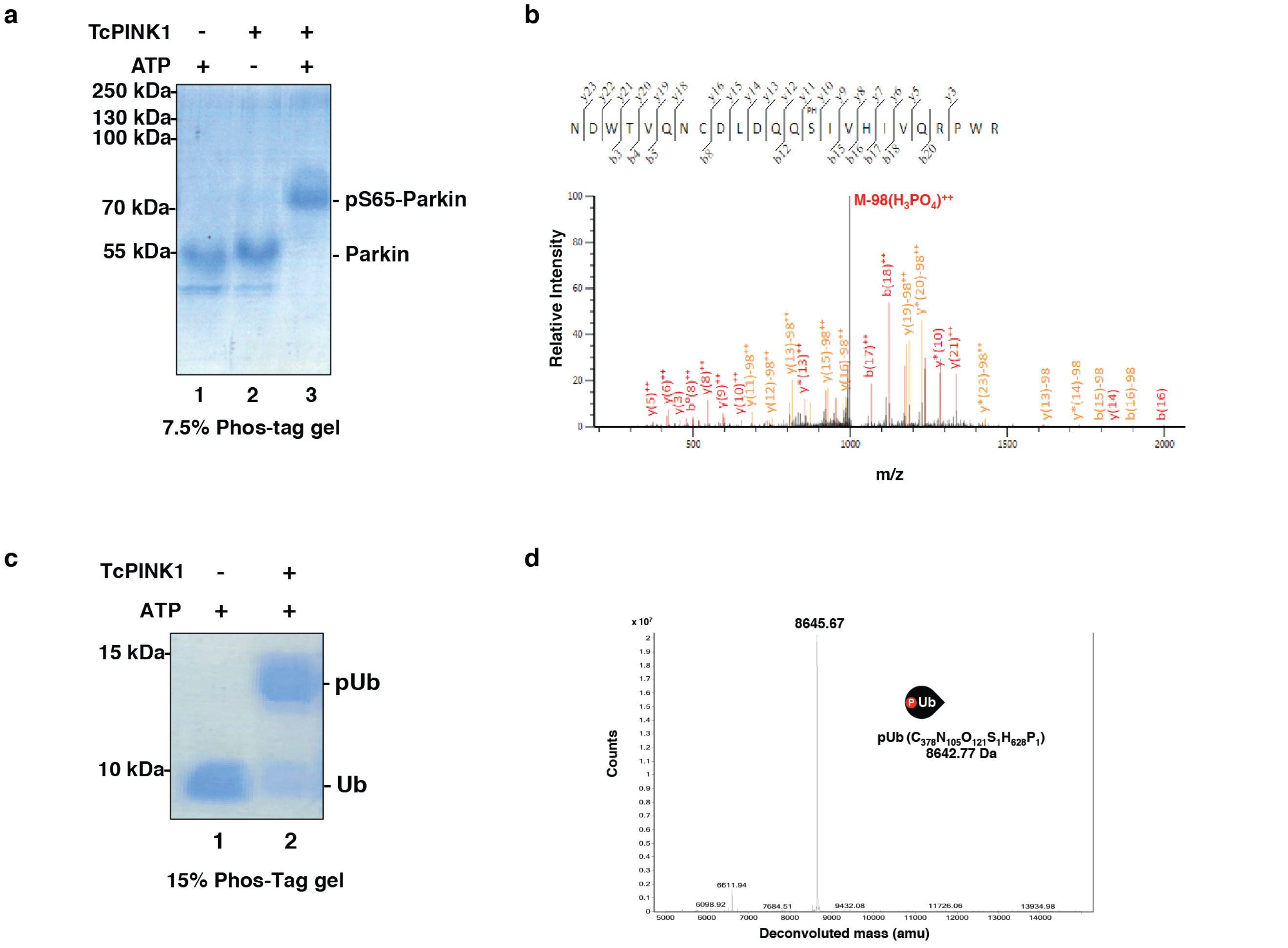

Supplementary Figure 3 | Characterization of Parkin and Ubiquitin phosphorylation. (**A**) Parkin is efficiently and specifically phosphorylated by PINK1 in the presence of ATP, but not PINK1 alone. The 7.5% SDS-PAGE gel was run using Phos-Tag (Wako Chemicals) and Coomasie-stained. (**B**) Identification of phosphorylation at Parkin S65. An MS/MS spectrum of PINK1-phosphorylated Parkin identifies the peptide fragment phosphorylated at Parkin S65. The major peak arises from neutral loss of H_3PO_4 from the intact peptide. (**C**) Ubiquitin is phosphorylated by PINK1. A 15% SDS-PAGE gel was run using Phos-Tag and Coomasie-stained. Phosphorylated Ub was subsequently purified by ion-exchange chromatography prior

to use. (**D**) MS spectrum of PINK1-phosphorylated Ubiquitin yields a deconvoluted mass (AMU) consistent with the theoretical mass of pUb (8644.7866).

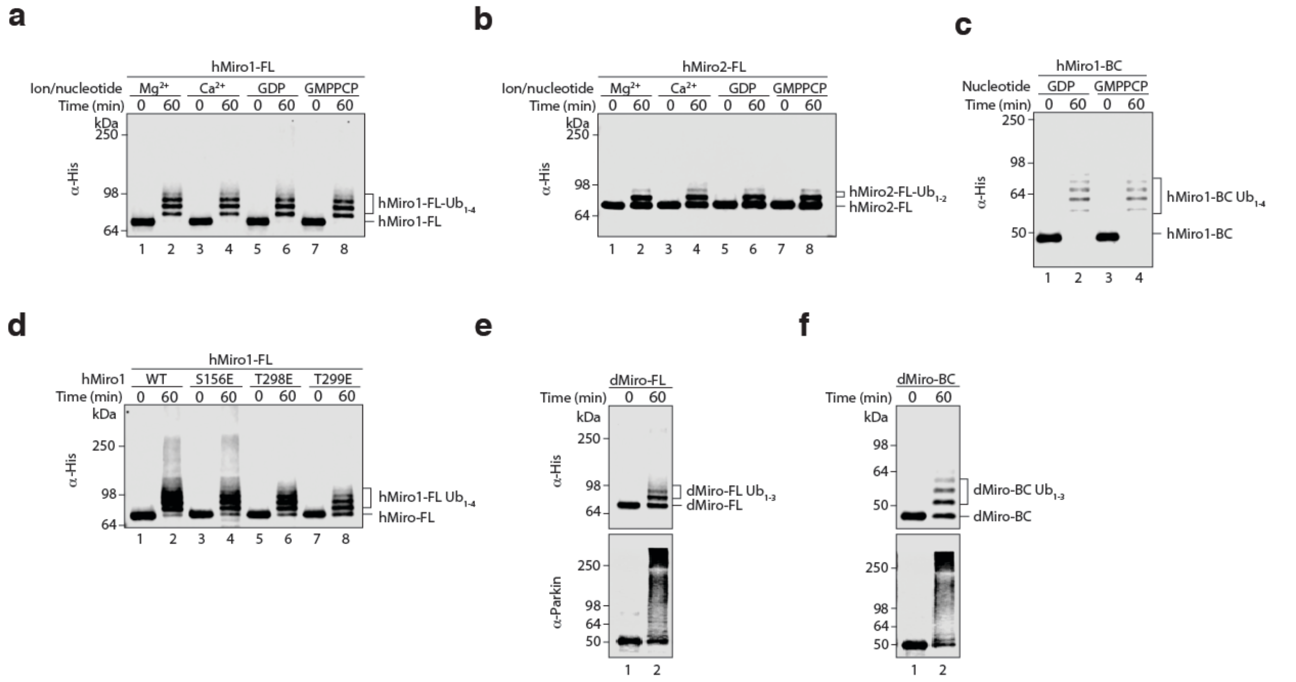

Supplementary Figure 4 | Ubiquitination of hMiro homologs is independent of ions, nucleotides, Miro phosphorylation and conserved in *Drosophila* Miro. (**a**,**b**) hMiro1-FL (a) or hMiro2-FL (b) ubiquitination is identical in the presence of either Mg^{2+}, Ca^{2+},GDP or the non-hydrolysable GTP analog GMPPCP. Ion/nucleotide exchange protocol is described in Methods. (**c**) For hMiro1-BC, which harbors just the cGTPase domain, ubiquitination is identical in the presence of either GDP or GMPPCP. (**d**) hMiro1-FL ubiquitination is not increased by either of three phosphomimetic mutations. Phosphomimetic mutations were introduced into hMiro1-FL at three sites previously identified to be targets of phosphorylation by PINK1 (S156, T298, and T299). hMiro1-FL S156E ubiquitination is indistinguishable from hMiro1-FL WT. The T298E and T299E mutations both have a mild inhibitory effect on hMiro1-FL ubiquitination. (**e**) Full length *Drosophila* dMiro-FL is ubiquitinated by human p-S65 Parkin with an efficiency resembling that of hMiro2-FL ubiquitination. (**f**) dMiro-BC is also ubiquitinated by human p-S65 Parkin.

a **K153**

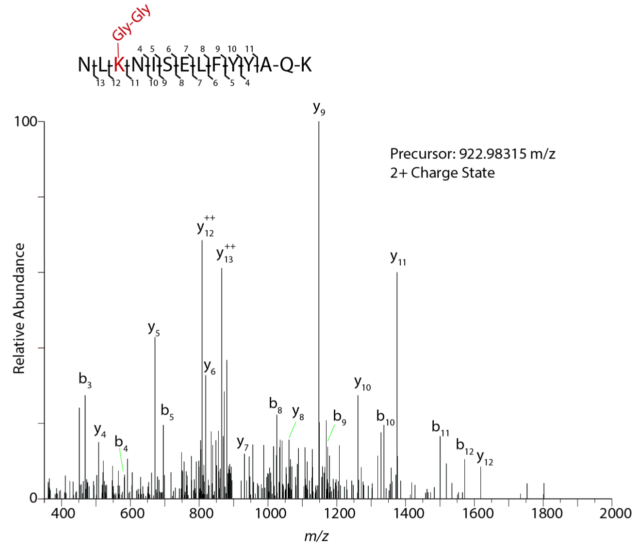

b **K235**

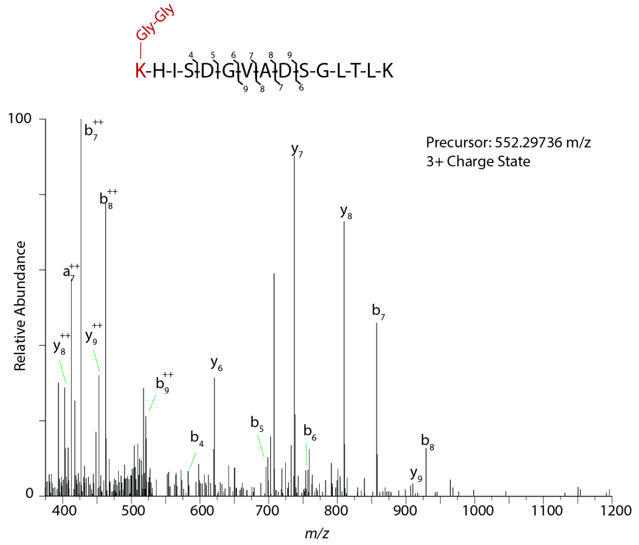

c **K572**

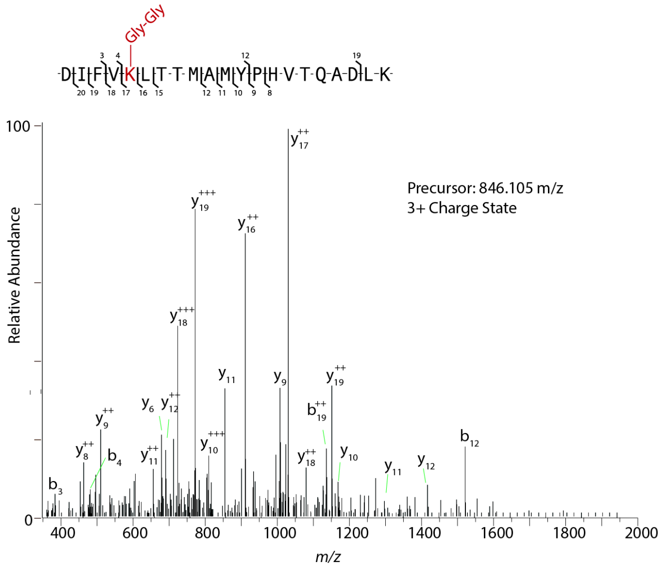

d **K96**

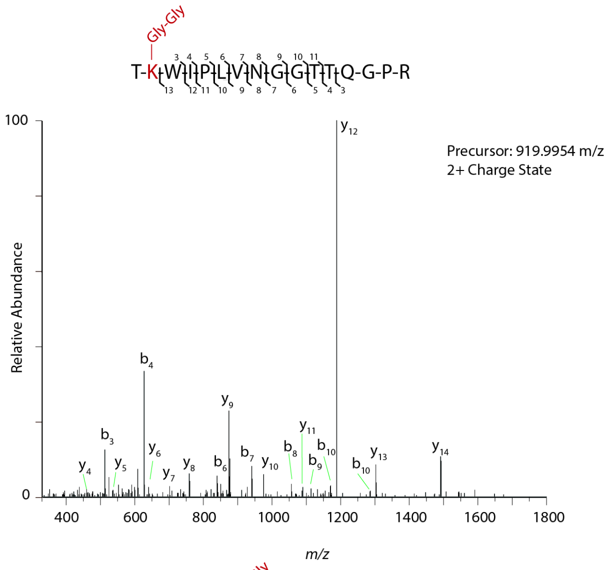

e **K119**

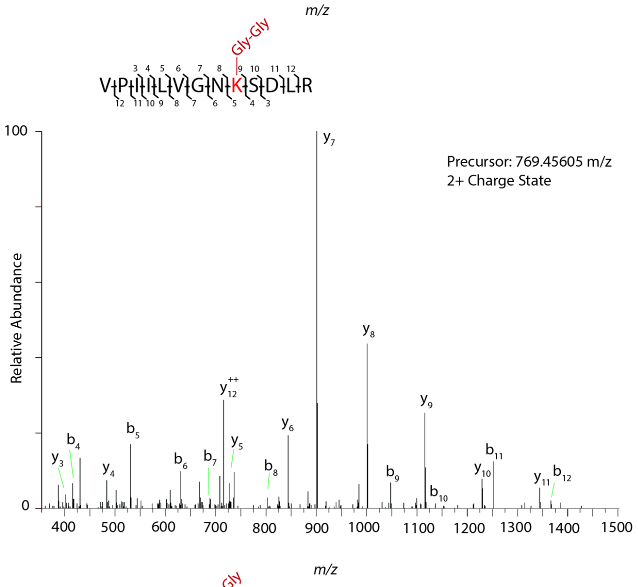

f **K164**

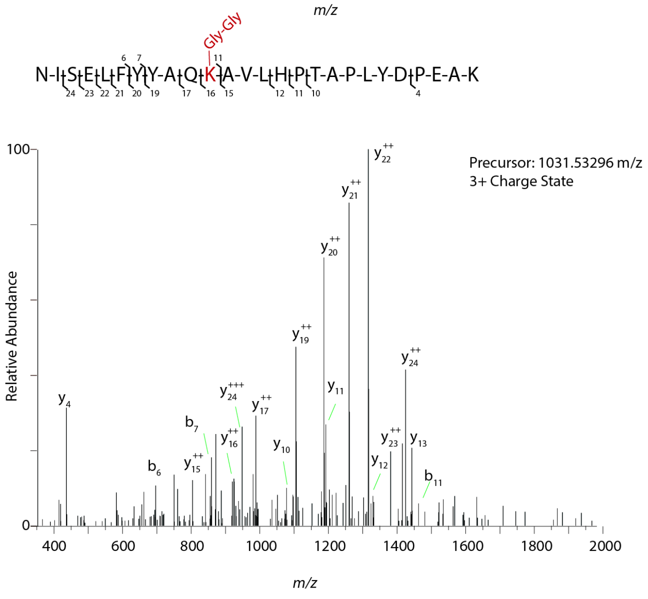

Supplementary Figure 5 | MS/MS spectra for diGly sites in hMiro1-FL and hMiro2-FL. (a-c) MS/MS spectra identifying hMiro1 lysines, K153 in the nGTPase, K235 in ELM1 (hEF1), and K572 in the cGTPase. **(d-f)** MS/MS spectra identifying hMiro2 lysines, K96, K119, and K164, all of which are in the nGTPase. All sites were identified with high confidence.

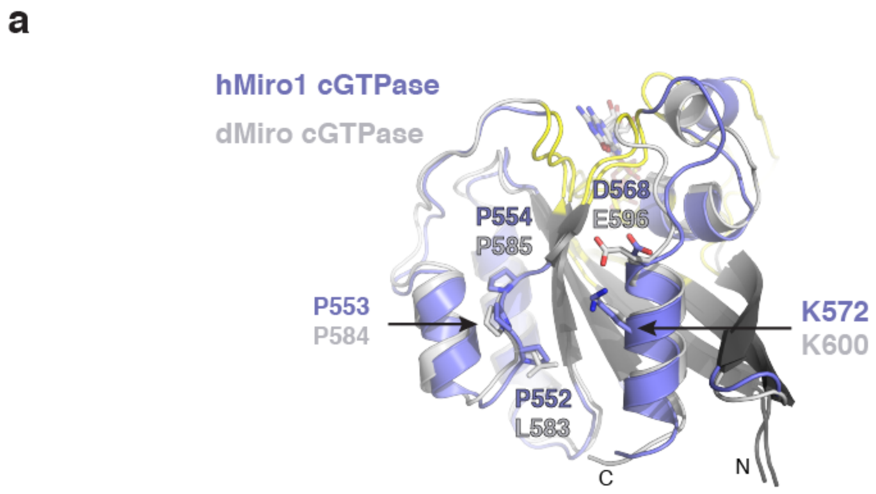

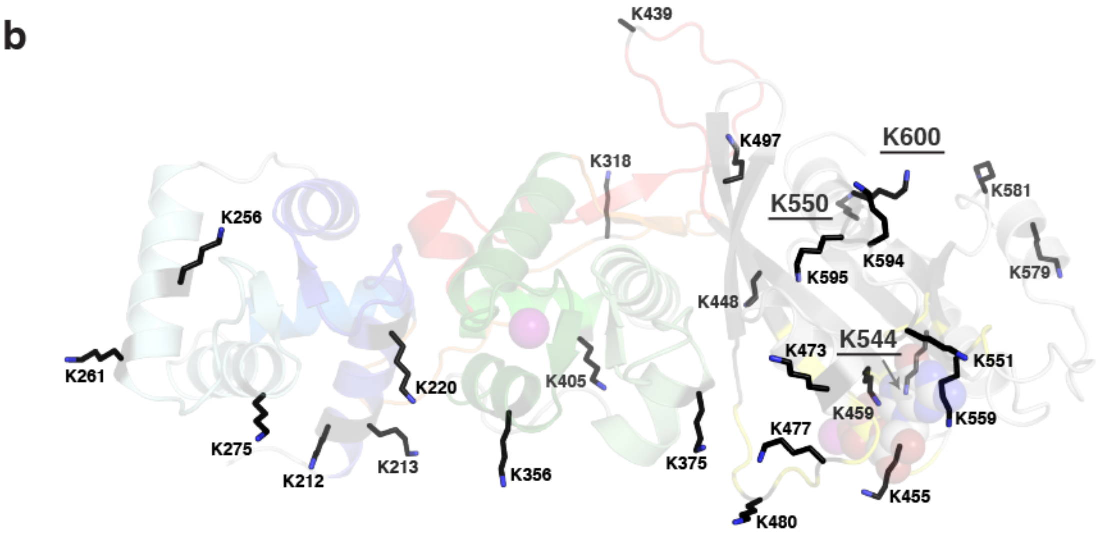

Supplementary Figure 6 | dMiro cGTPase structure comparison and lysine map. (**a**) The chemical environment of the dMiro K600 lysine is similar to that of the hMiro1 prioritized K572 lysine. Note the conserved proline-rich loop (to left), and the substitution of E596 for aspartate along the same face of the C-terminal alpha-helix, one turn about K572/K600. (**b**) Top view of

dMiro-BC cartoon shown in the background, with all lysines highlighted as sticks and labeled. Ubiquitinated lysines identified in this study (K544, K550, and K600) are underlined. Remaining lysines are labeled in black. Note that K600 in the dMiro cGTPase domain corresponds to K572 in the hMiro1 cGTPase domain, highlighting the conserved nature of this ubiquitination site.

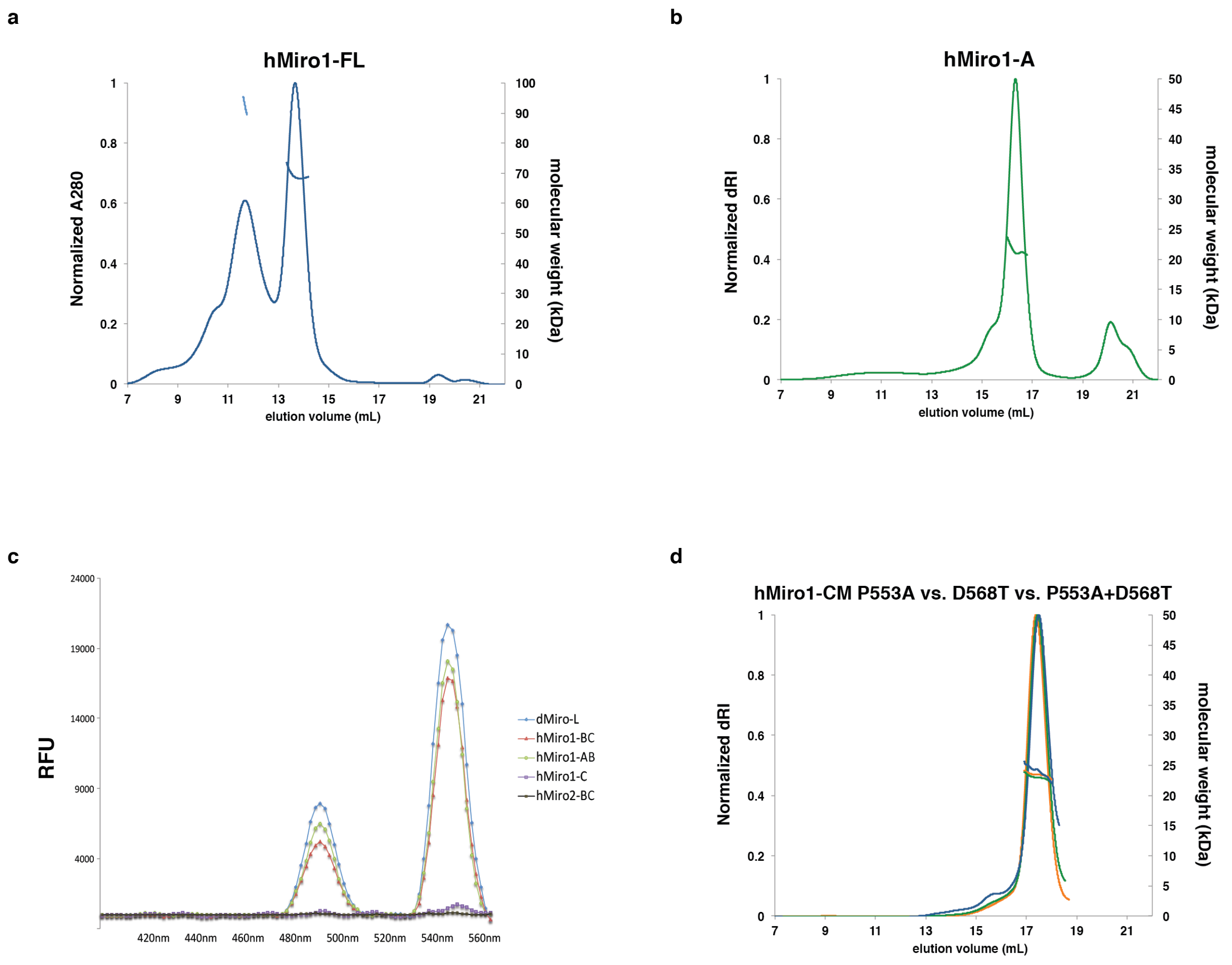

Supplementary Figure 7 | hMiro1 and its domain truncations/mutations are well-folded. **(a)** SEC-MALS trace of hMiro1-FL, which elutes at its expected molecular weight (66.7 kD). Note the UV peak at 11.5 mL corresponds to a contaminant. **(b)** SEC-MALS trace of hMiro1-A, which elutes at its expected molecular weight (21.3 kD). **(c)** Fluorescence emission-scan spectra revealing tryptophan-enhanced FRET stimulation of Tb^{3+} luminescence in hMiro1-AB. Tb^{3+} is a well-characterized probe of Ca^{2+}-binding sites; its excitation wavelengths overlap with the tryptophan emission band thereby allowing FRET excitation, albeit with a stringent Förster distance of ~2.5Å. hMiro1 and dMiro (but not hMiro2) contain a tryptophan sidechain ~5Å from

the cEF2 Ca^{2+} site, providing a mechanism to assay the presence of the intact cEF2 Ca^{2+}-binding site. Shown overlaid are spectra obtained from hMiro1-BC, hMiro1-AB, hMiro1-C, hMiro2-FL, and dMiro-FL. hMiro1-BC, hMiro1-AB, and dMiro-FL exhibit strong Tb^{3+} excitation, with characteristic emission bands at 487nm and 545nm; hMiro1-C (no Ca^{2+} site), and hMiro2 (no adjacent tryptophan) do not. **(d)** SEC-MALS traces of three hMiro1-C_M mutants (P553A, D568T, and P553A+D568T), which elute at approximately their expected molecular weights (21.9 kD). Note the dRI traces are truncated at ~ 19 mL due to a contaminating sucrose peak that elutes at the total column volume. All buffers contained Mg^{2+}. For SEC-MALS traces demonstrating the stability of other hMiro truncations/mutations, refer to Fig. 4 (hMiro1-C, hMiro1-CM, and hMiro2-C), and Fig. S2 (hMiro1-BC).

a

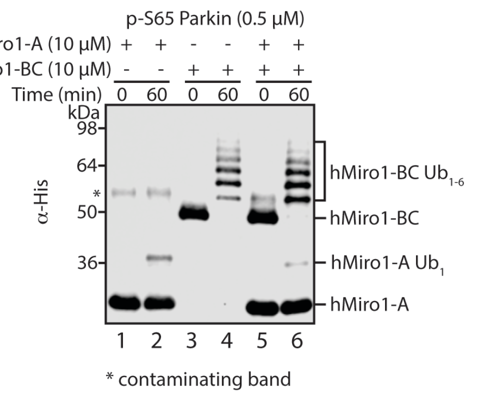

b

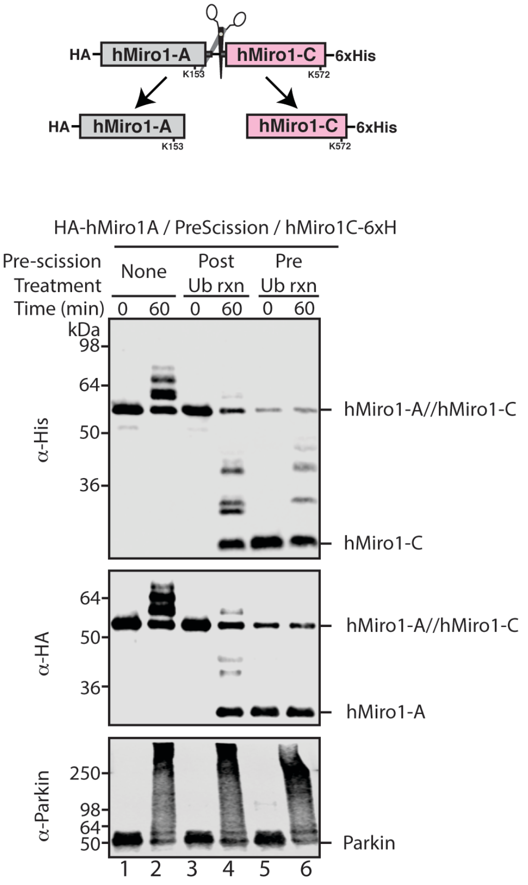

Supplementary Figure 8 | Inefficient hMiro1 nGTPase ubiquitination in the absence of native linkage with the hMiro1 EF hands and cGTPase domains. (**a**) The presence of HMiro-BC "in trans" does not enhance ubiquitination of hMiro1-A. hMiro1-A and hMiro1-BC (10 µM) were incubated individually or together in the presence of p-S65 Parkin (0.5 µM). hMiro1-A alone is only weakly ubiquitinated, while hMiro1-BC alone is robustly ubiquitinated, consistent with ubiquitination experiments at lower (1 µM) hMiro1 concentrations (Fig. 3). In the presence of hMiro1-BC, hMiro1-A ubiquitination is not enhanced. (**b**) Artificial fusion of the hMiro1 nGTPase to the cGTPase weakly enhances nGTPase ubiquitination. An N-terminally HA-tagged hMiro1 nGTPase (aa 1-180) was fused to a C-terminally 6xHis-tagged hMiro1

cGTPase (aa 411-592) via a linker consisting of the PreScission protease sequence (LEVLFQGPG). The HA-hMiro1-A/PreScission/hMiro1-C-6xHis fusion (1 μM) incubated with p-S65 Parkin (0.5 μM) generates a ubiquitin ladder (lanes 1-2). Cleavage with PreScission protease *after* the 60-minute ubiquitination reaction reveals low levels of ubiquitination of the nGTPase domain (lanes 3-4, α-HA), and more robust modification of the cGTPase domain (lanes 3-4, α-His). Cleavage with PreScission protease *before* the 60-minute ubiquitination reaction shows ubiquitination only of the cGTPase domain and not of the nGTPase (lanes 5-6). The α-Parkin blot serves as an internal positive control to confirm the presence of the PreScission protease does not interfere with the ubiquitination reaction.

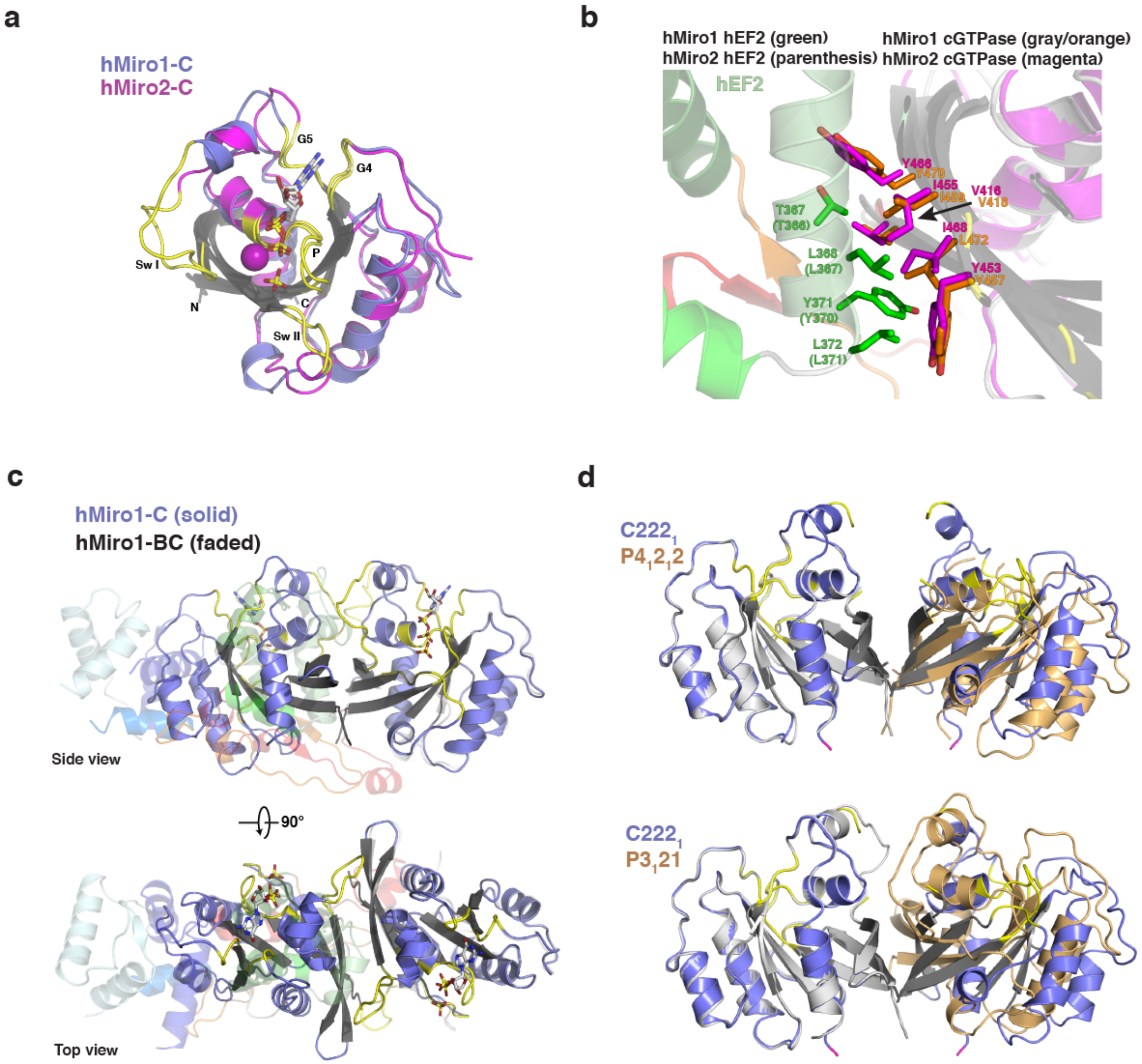

Supplementary Figure 9 | The hydrophobic hEF2/cGTPase interface mediates hMiro-C dimer formation. (**a**) Superposition of hMiro1-C (blue) and hMiro2-C (pink) highlights their similarity in overall GTPase fold, which is highly conserved over the core beta sheet and diverges primarily over the connecting loops (rmsd 0.78Å over 129 of 160 Cα atoms). Nucleotide-binding elements are shown in yellow and labeled as follows: P, P-loop; SwI,

Switch I; SwII, Switch II; G4-G5, G-protein nucleotide-binding motif 4-5. hMiro1-C is shown bound to GDP-Pi; hMiro2-C is shown bound to GDP. (**b**) Close-up view of the hMiro1 hEF2/cGTPase interface with hMiro2-C overlaid onto the hMiro1 cGTPase and interface residues highlighted in sticks. Note the nearly complete conservation of cGTPase hydrophobic residues mediating the hEF2 interaction (orange and pink sticks); these are the same residues that mediate hMiro-C dimerization (Fig. 4c,d). Although we were unable to obtain a structure of hMiro2-BC, sequence/structural comparison of hEF2 residues mediating the cGTPase interaction reveal complete conservation (green sticks). (**c**) The hEF2/cGTPase interface is mutually exclusive with cGTPase dimerization. Overlay of hMiro1-BC (multi-color, ghosted) and hMiro1-C (blue, solid) aligned by their cGTPase domains shows the cGTPase protomer partner in the dimer structure overlaps completely and exclusively with ELM2 (green). The hMiro1-C structure shown is from spacegroup $P3_121$. The side-view is the same as in Fig. 1b (hMiro1-BC) and Fig. 4a (hMiro1-C). (**d**) The cGTPase dimer observed in three different crystal environments. The hMiro1 crystallized in three different space groups, such that the two-fold symmetric dimer in each case exhibited crystallographic two-fold symmetry but each within distinct crystal packing environments. Shown are the overlays of two dimer pairs from three space groups obtained following superposition of one monomer (at left in each image). Note that the dimer interface exhibited some plasticity, characteristic of 'oily' packing across hydrophobic interfaces.

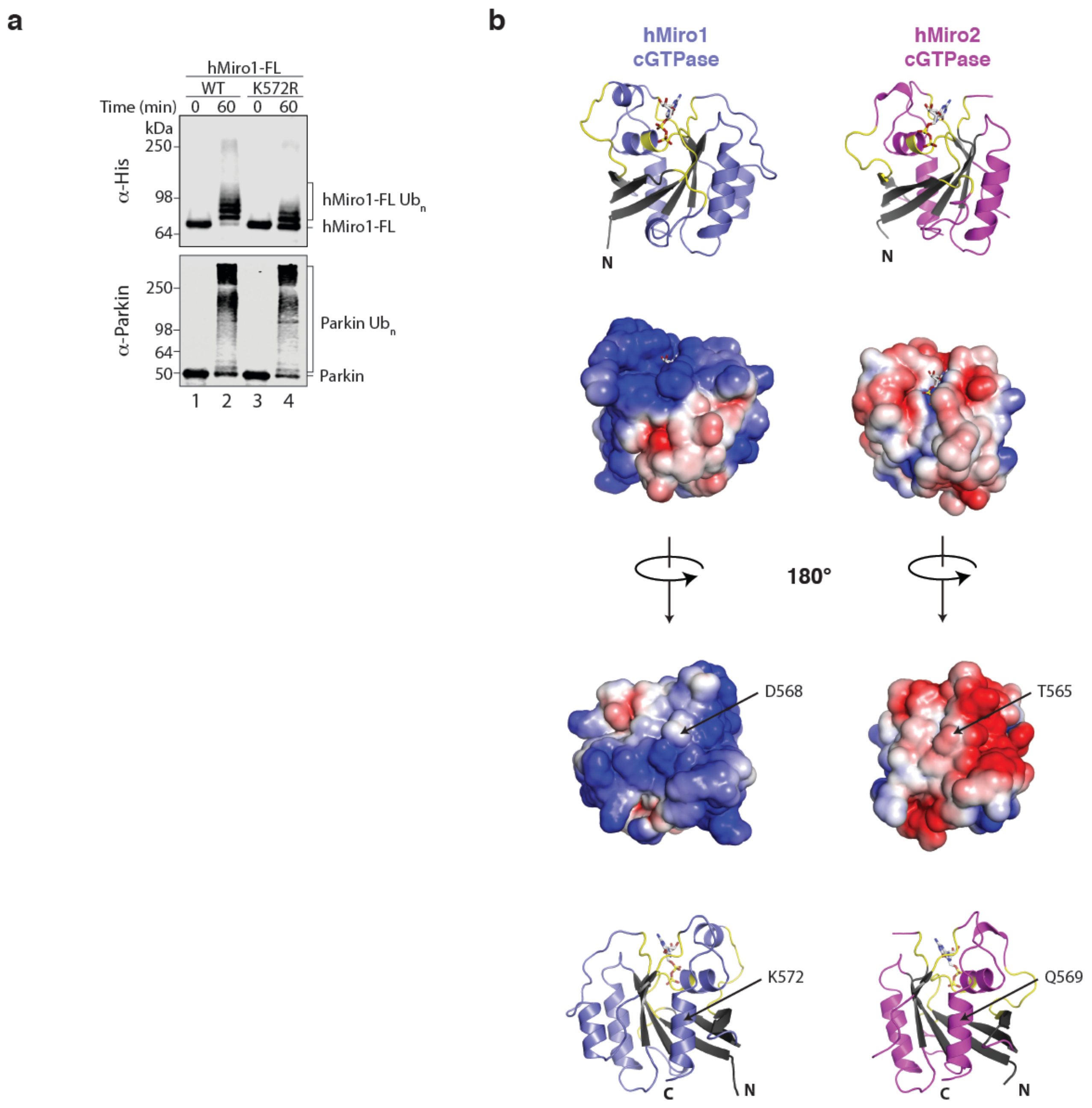

Supplementary Figure 10 | Differences in the hMiro cGTPases largely account for the difference in ubiquitination efficiency between hMiro1 and hMiro2. (**a**) The presence of the preferred K572 lysine in hMiro1 is primarily responsible for the higher ubiquitination

efficiency of hMiro1 as compared to hMiro2. hMiro1-FL K572R has decreased ubiquitination efficiency as compared with hMiro1-FL, reminiscent of ubiquitination levels of hMiro2, which lacks a preferred K572 equivalent. (**b**) Electrostatic surface potential map of hMiro1 and hMiro2 cGTPase domains. The hMiro1 cGTPase is quite positively charged (theoretical pI 8.9), while the hMiro2 cGTPase is negatively charged (theoretical pI 6.0), especially in the vicinity of the C-terminal helix harboring hMiro1 K572. The surface is colored according to the electrostatic potential at 298 K (−3 to 3 kT/e), with negative potential in red and positive potential in blue.

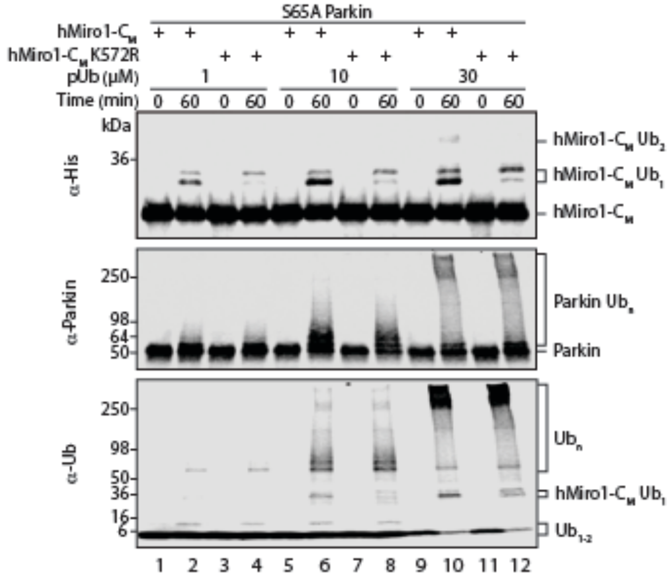

Supplementary Figure 11 | Conjugatable pUb activates unphosphorylated Parkin accompanied by substrate lysine prioritization. pUb activates unphosphorylated Parkin for substrate lysine prioritization. Comparison of hMiro1-C_M and hMiro1-C_M K572R substrate ubiquitination at three different pUb concentrations shows that S65A Parkin yields an hMiro1-C_M Ub_1 doublet consistent with K572 prioritization.

a

Miro2		R425C: MAF 0.33	
Human	405	LDQEKGQTQRSVLLCKVVGARGVGKSAFLQAFLGRGLGHQD	445
Mouse		LDQEKGQTQRSVLMCKVLGARGVGKSAFLQAFLGNSLGEAR	
Rat		LDQEKGQTQRSVLMCKVLGARGVGKSAFLQAFLGHSLGEAR	
Pig		AGPGKGQTQRNVLLCKVLGARGVGKSSFLRAFLGRGLGDAR	
Bovine		LDQEKGQTQRNVLLCKVVGARGVGKSSFLRAFLGHSLGHQ-	
Chicken		IDLEKGQTQRNVFLCKVLGARGAGKSAFLQAFLGRSLAAQR	
Xenopus		IDLEKGQTQRNVFLCRVIGPRGTGKSAFLRAFLGQSLEEQQ	
Zebrafish		LDLDNRQTQRTVFLCKVIGPRGTGKTDFLRAFLQRSTERSD	

b

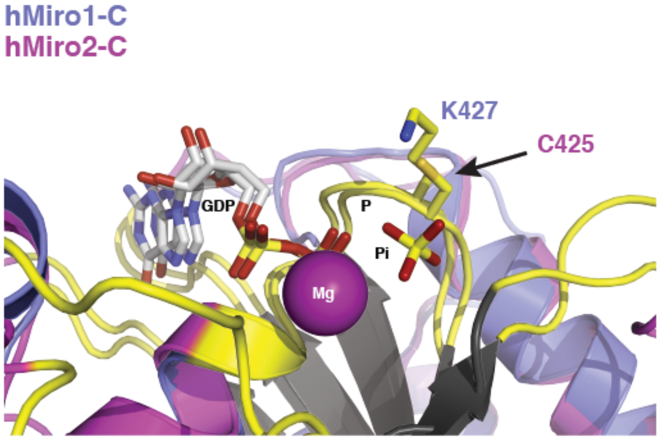

c

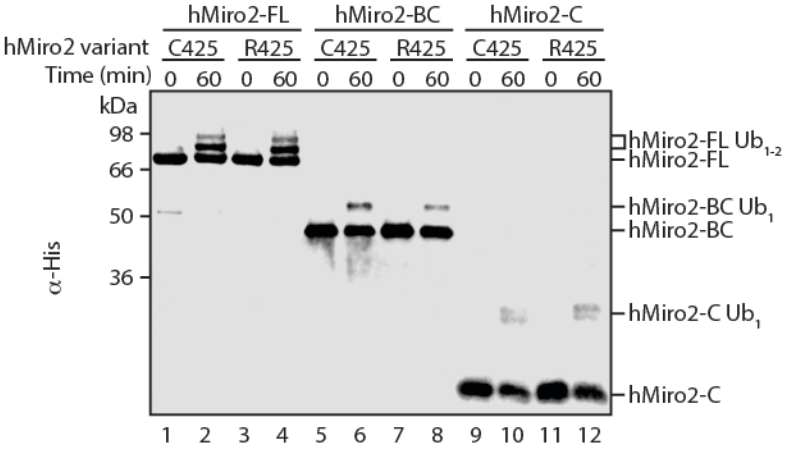

Supplementary Figure 12 | The common hMiro2 missense mutation R425C is neutral with respect to Parkin ubiquitination. (**a**) hMiro2 R425 is highly conserved across vertebrates with two Miro homologs. Sequence alignment of vertebrate Miro2 in the vicinity of hMiro2 R425 (highlighted in red). The minor allelic frequency (MAF) of the hMiro2 R425C missense mutation is 0.33 (ensembl rs3177338). (**b**) Structural comparison of the hMiro1 and hMiro2 cGTPase domains highlighting the C425 in the hMiro2 P-loop and the corresponding K427 in hMiro1. Note the proximity to the nucleotide – hMiro1 K427 is within hydrogen bonding distance of the phosphate in the nucleotide binding pocket. (**c**) Side-by-side comparison of hMiro2-FL, hMiro2-BC, and hMiro2-C R425 and C425 variants shows no difference in ubiquitination.
